# Single-cell transcriptomic profiling unravels the adenoma-initiation role of protein tyrosine kinases during colorectal tumorigenesis

**DOI:** 10.1038/s41392-022-00881-8

**Published:** 2022-02-28

**Authors:** Xiaobo Zheng, Jinen Song, Chune Yu, Zongguang Zhou, Xiaowei Liu, Jing Yu, Guangchao Xu, Jiqiao Yang, Xiujing He, Xin Bai, Ya Luo, Yu Bao, Huifang Li, Lie Yang, Mingqing Xu, Nan Song, Xiaodong Su, Jie Xu, Xuelei Ma, Hubing Shi

**Affiliations:** 1 Laboratory of Integrative Medicine, Clinical Research Center for Breast, State Key Laboratory of Biotherapy, West China Hospital, Sichuan University and Collaborative Innovation Center, 610041 Chengdu, Sichuan China; 2 Frontier Science Center for Disease Molecular Network, West China Hospital, Sichuan University, 610041 Chengdu, Sichuan China; 3 Institute of Digestive Surgery, Sichuan University, and Department of Gastrointestinal Surgery, West China Hospital, West China School of Medicine, Sichuan University, 610041 Chengdu, Sichuan China; 4 Research Core Facility, West China Hospital, Sichuan University, 610041 Chengdu, Sichuan China; 5 Department of Gastrointestinal Surgery, State Key Laboratory of Biotherapy and Cancer Center, West China Hospital, Sichuan University, and Collaborative Innovation Center for Biotherapy, 610041 Chengdu, Sichuan China; 6 Department of Liver Surgery, West China Hospital, Sichuan University, 610041 Chengdu, Sichuan China; 7 Beijing Institute of Tropical Medicine, Beijing Friendship Hospital, Capital Medical University, 100050 Beijing, China; 8 Biomedical Pioneering Innovation Center (BIOPIC), and State Key Laboratory of Protein and Plant Gene Research, Peking University, 100871 Beijing, China; 9 Institutes of Biological Sciences, Zhongshan-Xuhui Hospital, Fudan University, 200032 Shanghai, China; 10 Department of biotherapy, State Key Laboratory of Biotherapy, Cancer Center, West China Hospital, Sichuan University, 610041 Chengdu, Sichuan China

**Keywords:** Gastrointestinal cancer, Oncogenes, Cancer genetics

## Abstract

The adenoma-carcinoma sequence is a well-accepted roadmap for the development of sporadic colorectal cancer. However, cellular heterogeneity in aberrant epithelial cells limits our understanding of carcinogenesis in colorectal tissues. Here, we performed a single-cell RNA sequencing survey of 54,788 cells from patient-matched tissue samples, including blood, normal tissue, para-cancer, polyp, and colorectal cancer. At each stage of carcinogenesis, we characterized cell types, transcriptional signatures, and differentially expressed genes of distinct cell populations. The molecular signatures of epithelial cells at normal, benign, and malignant stages were defined at the single-cell scale. Adenoma and carcinoma precursor cell populations were identified and characterized followed by validation with large cohort biopsies. Protein tyrosine kinases (PTKs) BMX and HCK were identified as potential drivers of adenoma initiation. Specific BMX and HCK upregulations were observed in adenoma precursor cell populations from normal and adenoma biopsies. Overexpression of BMX and HCK significantly promoted colorectal epithelial cell proliferation. Importantly, in the organoid culture system, BMX and HCK upregulations resulted in the formation of multilayered polyp-like buds protruding towards the organoid lumen, mimicking the pathological polyp morphology often observed in colorectal cancer. Molecular mechanism analysis revealed that upregulation of BMX or HCK activated the JAK-STAT pathway. In conclusion, our work improved the current knowledge regarding colorectal epithelial evolution during carcinogenesis at the single-cell resolution. These findings may lead to improvements in colorectal cancer diagnosis and treatment.

## Introduction

Colorectal cancer (CRC) is the third most common cancer worldwide, with about 1.8 million new cases and 880,800 deaths per year.^1^ Among all subtypes, sporadic (or nonhereditary) types account for about 65% of new cases of CRC.^2^ The adenoma-carcinoma sequence is the established model for sporadic CRC development, where adenomas are considered as major precancerous lesions that may lead to CRC development.^3–6^ We now have a comprehensive view of the adenoma-carcinoma sequence from the perspectives of genomics, epigenetics, transcriptomics, and proteomics.^7–11^ In terms of CRC pathogenesis, we know that ~70% of adenomas carry APC mutations.^12^ Carcinogenesis is triggered through activating mutations of KRAS (or BRAF) oncogene and inactivating mutations of the TP53 tumor suppressor gene.^7,9^ These aberrant genetic alterations are often accompanied by high microsatellite instability due to methylation of MLH1 gene promoter.^10^

Although these molecular mechanisms provide a guideline for CRC treatment and prognosis, we still have not identified the driver events for initiation of adenoma in precursor cell populations. Understanding adenoma initiation will provide clues for CRC prevention, diagnosis, and therapy. The heterogeneity of intra-tumoral malignant cells is a key feature of tumor biology, and clarifying the traits of individual populations will improve treatment response and patient survival.^13,14^ However, traditional molecular profiling largely relied on Sanger sequencing or bulk-tissue analysis, obscuring the signatures of distinct cell populations within adenomas and carcinomas. To improve the status quo, it is essential to comprehensively describe phenotypes and stages of cell subsets during colorectal carcinogenesis.

Advances in single-cell RNA sequencing (scRNA-seq) have revolutionized our capability to characterize transcriptomes in thousands of individual cells, enabling an unbiased and in-depth analysis of multiple cell populations within tumors.^15–19^ Recently, scRNA-seq was successfully applied to characterize CRC transcriptomic signatures.^20–25^ In colon cancer tissues, scRNA-seq and T-cell receptor sequencing were used to analyze distinct myeloid populations and relationships among T-cell sub-populations.^20,24^ The genomic and transcriptomic evolution of a hereditary CRC subtype, familial adenomatous polyposis, was determined at the single-cell scale, providing insight into the evolutionary mechanism leading to the disease progression.^22^ In addition, single-cell omics technologies have been applied to explore diversity and genomic evolution in CRC carcinogenesis/metastasis.^21,23,25^ However, these studies mainly focused on cell populations within carcinoma. Therefore, we know little about single-cell transcriptomes in adenoma or about the dynamic evolution of each cell type during the adenoma-carcinoma sequence.

Inter-individual variations, including genetic background, diet, and gut microbiota, may influence the conclusions drawn from investigating epithelial evolution during colorectal tumorigenesis. To avoid this problem, in our study, we simultaneously collected biopsies of clinical lesions representing normal tissue, benign adenoma, and malignant tumor from the same patient. Of note, these lesions represent sequential stages in colorectal carcinogenesis and are ideal models for tracing CRC evolution. Here, we performed a scRNA-seq survey of 54,788 cells from 12 samples of four CRC patients and constructed a single-cell transcriptomic atlas of CRC evolution. Based on the atlas, we characterized the molecular signatures of epithelial cell population during colorectal carcinogenesis at the single-cell scale, then identified and validated driver genes in intermediate populations within the normal-adenoma-carcinoma sequence.

## Results

### Single-cell transcriptomic profiling of colorectal normal, adenoma, and carcinoma tissues

**Fig. 1:**
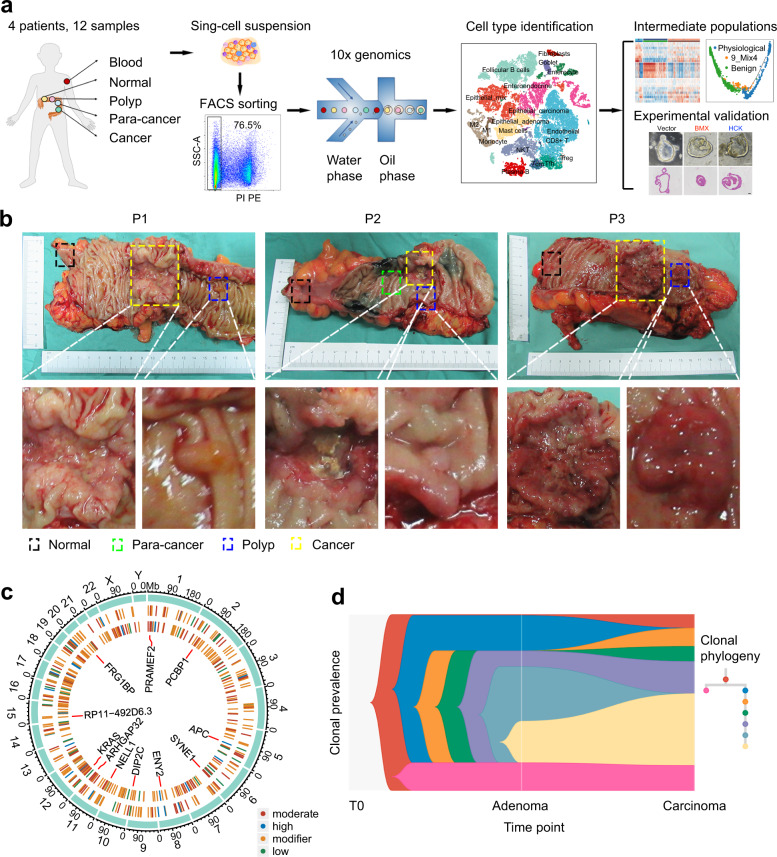
Sample collection and quality control. **a** Flowchart of library preparation and data analysis. **b** Samples were collected from surgically resected specimens of patients with colorectal polyp and cancer. Patients were numbered as patient 1 (P1), patient 2 (P2), and patient 3 (P3). **c** Circos graph shows single nucleotide variants in adenoma and carcinoma (exemplified by P3). The inner and intermediate cycles represent carcinoma and adenoma, respectively. Colors represent variant impact. The same single nucleotide variants of adenoma and carcinoma are shown in the center of the cycle. **d** Clonal evolution of adenoma and carcinoma (exemplified by P3)

To dynamically dissect the evolution and molecular signatures of each cell type during the normal-adenoma-carcinoma sequence, we profiled the transcriptome of each cell population using scRNA-seq technology (Fig. 1a). We concurrently collected samples of blood, normal, para-cancer, polyp, and cancer tissues from the same patient who underwent radical surgeries (Fig. 1b). Twelve tissue samples were collected from four untreated CRC patients with microsatellite stability (Supplementary Table 1). We confirmed the pathological conditions (i.e., adenoma and carcinoma) by H&E staining (Supplementary Fig. 1a). Through somatic short-variant analysis, we consistently observed multiple mutations in patient-matched adenoma and carcinoma (Fig. 1c). This genetic similarity highly implies an inherited lineage correlation among normal tissue, adenoma, and carcinoma. Clonal phylogeny analysis^26,27^ then identified the dynamic alterations of cell populations (Fig. 1d), further confirming the evolutionary relationship. These quality checks showed that biopsies from patient-matched sequential lesions recapitulated the pathological progress of colorectal tumorigenesis.

**Fig. 2:**
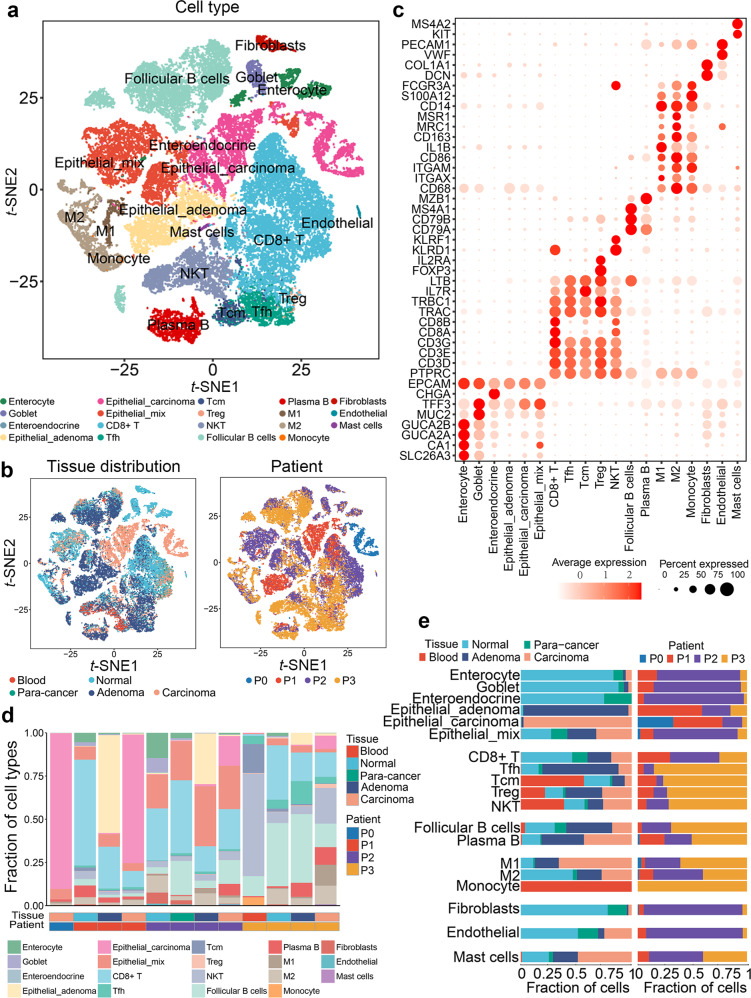
Cell type constitution of colorectal normal, para-cancer, adenoma, and carcinoma tissues. **a** *t*-SNE plots of cells from four patients (12 samples). Colors represent cell types. Cells were clustered into 19 sub-clusters based on biological annotation. Each dot represents a single cell. **b** *t*-SNE plots of tissue origin (left) and patient origin (right). Each dot represents a cell, and colors correspond to cell origins. **c** Log-normalized expression levels of canonical marker genes for the above 19 cell types. Circle size represents the percentage of cells that express the gene, and colors represent the average expression value within a cluster. **d** Bar plot showing the fraction of each cell type in 12 samples, with rows representing cell types and columns representing samples. **e** Bar plot presenting the fraction of tissue origin (left) and the fraction of patient origin (right) for the 19 cell types. Colors represent cell origins

The single-cell suspension for establishing a sequencing library was prepared by digesting fresh tissues with trypsin. Viable cells were obtained by flow cytometry to sort propidium iodide-negative events (Supplementary Fig. 1b). We obtained transcriptomes of 54,788 individual cells by performing scRNA-seq on the droplet-based 10× Genomics platform (Supplementary Table 2). Following a strict quality filtering process, we used the Scimpute^28^ algorithm to impute the dropouts due to excess zero counts of transcripts captured within individual cells in the scRNA-seq data. Then, principal component analysis (PCA) was performed on variably expressed genes, and unsupervised graph-based clustering was performed to classify the cells using the Seurat^29^ algorithm. The cell populations were visualized using *t*-SNE plots based on the cluster, tissue origin, and patient origin (Fig. 2a, b). Based on several canonical marker genes for known cell lineages, identified clusters were annotated to biological cell types: enterocytes (*GUCA2A*), goblet (*MUC2*), enteroendocrine (*CHGA*), adenoma epithelial (epithelial cells derived from adenoma), carcinoma epithelial (epithelial cells derived from carcinoma), mixed epithelial (epithelial cells derived from heterogeneous tissue), CD8^+^ T (*CD8A*), follicular helper T (*CXCR5*), central memory T (*IL7R*), regulatory T (*FOXP3*), natural killer T (*KLRD1*), follicular B (*MS4A1*), plasma B (*MZB1*), M1 macrophage (*IL1B*), M2 macrophage (*CD163*), monocyte (*CD14*), fibroblasts (*COL1A1*), endothelial (*VWF*), and mast (*KIT*) cells (Fig. 2c and Supplementary Fig. 2a, b). Two independent approaches were applied to exclude the influence of batch effects. First, the enterocytes and goblet cells from normal intestinal tissues were defined by combo marker genes *GUCA2A*-*CA1*-*SLC26A3*, and *MUC2*,^30^ respectively (Supplementary Fig. 3a–c). We observed that the enterocytes and goblet cells origin from all three patients merged together on the *t*-SNE plot respectively, highlighting an epithelial subtype-specific manner (Supplementary Fig. 3d). In addition, the harmony algorithm was applied to ensure that batch variation among patients was marginal (Supplementary Fig. 3e, f). Hence, all these results ensured that our scRNA-seq data met the requirement of quality, which was suitable for further data mining.

Most cell populations were observed in all tissues and patients despite individual differences (Fig. 2d and Supplementary Fig. 3g). Normal epithelial cells from different patients grouped together, meaning that they shared similar expression signatures. In contrast, aberrant epithelial cells (in adenoma or carcinoma) from different patients clustered separately and displayed highly heterogeneous characters, indicating diversified molecular mechanisms of carcinogenesis (Fig. 2e). We performed inferCNV analysis on epithelial cells extracted from normal, adenoma, and carcinoma tissues (Supplementary Fig. 4a, b). The results showed that normal tissue-derived epithelial cells harbored few deletions or amplifications compared with adenoma- and carcinoma-derived epithelial cells. A slight increase of copy number variations (CNVs) was observed in the epithelial cells from adenoma and was obviously accumulated in the epithelial cells from carcinoma. Notably, the adenoma and carcinoma tissues shared a series of common CNVs at multiple chromatin loci, indicating an evolutionary relationship. Interestingly, a total of 17 circulating tumor cells (CTCs) were identified in a blood sample from patient 3 (Supplementary Fig. 3g) using canonical epithelial cell marker genes (Supplementary Fig. 5a) and large-scale CNV analysis (Supplementary Fig. 5b). Differentially expressed genes (DEGs) analysis showed a distinct transcriptional character in the CTCs although they shared some tumor-specific marker genes (such as *TGFB1* and *SOX9*) with tumor cells in solid lesions (Supplementary Fig. 5c). Further gene set variant analysis (GSVA) analysis identified a series of CTC-specific signatures related to platelet activation, regulation of cell death, and cell adhesion, suggesting that these pathways might be involved in the pathological process of CTCs (Supplementary Fig. 5d). All these data characterized the cellular population constitution in the indicated stages of CRC evolution.

### Three epithelial cell stages during the evolution of colorectal carcinogenesis

**Fig. 3:**
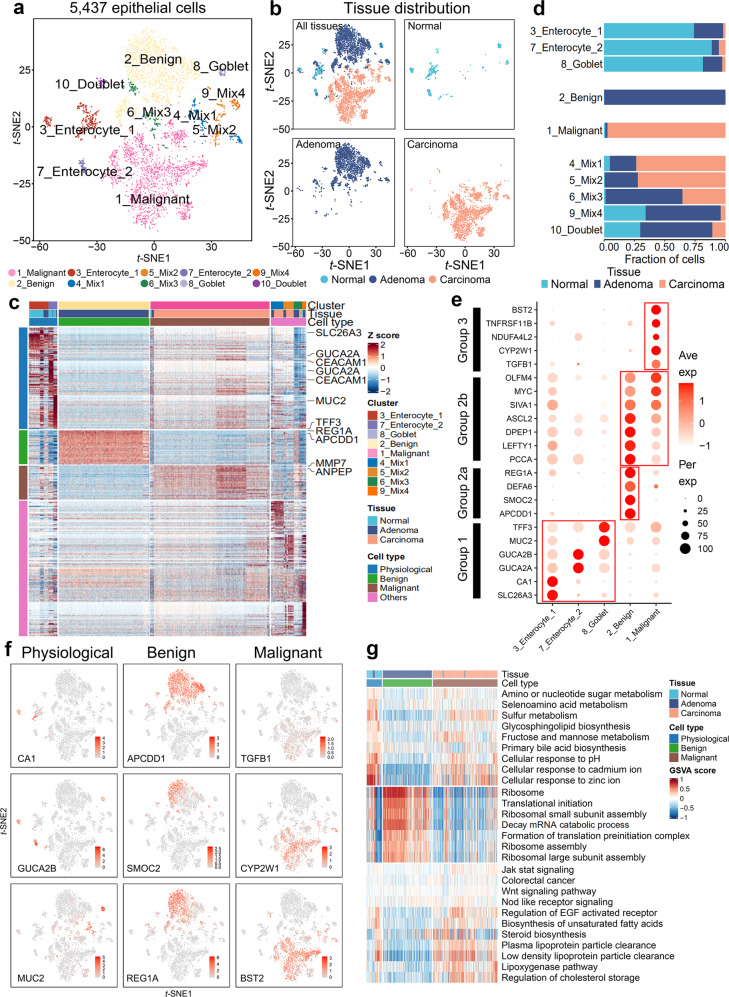
Three typical stages of epithelial cells during colorectal carcinogenesis and transcriptomic characterization. **a** *t*-SNE plot of 5,437 epithelial cells from P1 (3 samples). These cells were defined as 10 cell sub-clusters based on biological annotation and inferCNV analysis. Each dot represents a single cell, and colors correspond to cell types. **b** *t*-SNE plot of P1 epithelial cells and the three faceted *t*-SNE plots (normal, adenoma, and carcinoma). Colors represent the tissue origin of cells. **c** Top 50 differentially expressed marker genes in each cluster. Columns denote cells annotated with cell type, tissue, and cluster. Normal epithelial cell clusters (3_Enterocyte_1, 7_Enterocyte_2, and 8_goblet) were set as physiological cells. Rows indicate genes annotated with cell type and exemplar gene names, and colors represent *Z*-Score of log-normalized data. **d** Bar plot of tissue fraction in each cell sub-cluster. **e** Dot plot annotating physiological, benign, and malignant cell clusters by the expression of cluster-specific genes. Columns indicate cell sub-cluster. Circle size represents the percentage of cells that express the gene, and colors represent the average expression value within a cluster. **f** Expression of key marker genes in physiological (*CA1, GUCA2B, MUC2*), benign (*APCDD1, SMOC2, REG1A*), and malignant (*TGFB1, CYP2W1, BST2*) cells. Colors represent the log-normalized data. **g** GSVA results for physiological, benign, and malignant cells. Rows represent gene sets, and columns represent cells annotated with cell type and tissue. Colors indicate the GSVA score for each cell

**Fig. 4:**
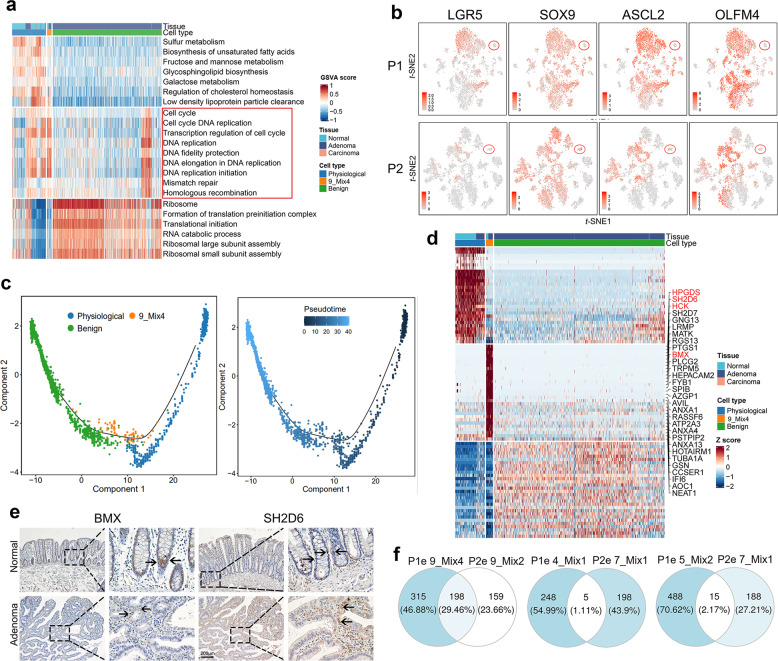
Intermediate stages of epithelial cells among the physiological, benign, and malignant stages. **a** Signature scores for cells from physiological, benign, and mixed clusters (9_Mix4) in P1. Columns represent cells (*n* = 2420) annotated with cell type and tissue. Colors represent *Z* scores. **b** Log-normalized expression levels of key marker genes of intestinal stem and base crypt cells (*LGR5, SOX9, ASCL2, OLFM4*). Red cycle indicates cells of cluster 9_Mix4 (top, P1, *n* = 73) and cluster 9_Mix2 (bottom, P2, *n* = 87). **c** Single-cell trajectory of 554 physiological epithelial cells, 73 adenoma-precursor cells (cluster 9_Mix4), and 1793 benign epithelial cells. The trajectory was constructed using monocle according to gene expression. Each dot represents a single cell, and colors represent cell types (left) and the pseudotime of trajectory (right). **d** Heatmap of differentially expressed genes among normal, adenoma precursor (cluster 9_Mix4), and adenoma epithelial cells. Genes related to carcinogenesis are highlighted. **e** Representative images of BMX and SH2D6 IHC staining of normal tissue (*n* = 20) and adenoma (*n* = 20). Black arrows indicate positively stained cells. Scale bar, 200 μm. **f** Venn diagrams show the overlap of upregulated genes in P1 epithelial mixed clusters (cluster 4, 5, and 9) and P2 epithelial mixed clusters (cluster 7 and 9)

To better understand colorectal carcinogenesis, we focused on epithelial cells in normal, adenoma, and carcinoma tissues. Considering the individual differences, we separately enrolled epithelial cells from the same patient to explore epithelial cell evolution. The epithelial cells from P1 and P2, as identified in Fig. 2a, were categorized based on the malignant degree using the inferCNV analysis, and were replotted using the *t*-SNE dimensional analysis (Fig. 3a and Supplementary Fig. 6a). Owing to the insufficient number of epithelial cells, we excluded patient 3 from this analysis (Supplementary Fig. 3g). Cells from normal, adenoma, and carcinoma tissues were clearly separated from each other (Fig. 3b and Supplementary Fig. 6b). We then categorized epithelial cells into physiological, benign, malignant, and other populations based on their pathological genetic markers^31^ (Fig. 3c and Supplementary Fig. 6c). Several intestinal cell subtypes were identified based on the canonical colorectal epithelial marker genes, including enterocytes (*GUCA2A*, *SLC26A3*, and *CA1*), goblet cells (*MUC2* and *TFF3*), enteroendocrine cells (*CHGA*), and stem cells (*LGR5*). The results of cell counting indicated that physiological, benign, and malignant cells were predominantly from normal, adenoma, and carcinoma tissues, respectively (Fig. 3d and Supplementary Fig. 6d). Representative DEGs were divided into three groups (1, 2, and 3) that were consistent with canonical markers, corresponding to physiological, benign, and malignant stages (Fig. 3e). Sequential upregulation of these DEGs during CRC development highlighted a temporal mechanism that drives the transition from normal to benign to malignant colorectal tissues. Notably, both benign and malignant populations contained a group of upregulated genes (group 2b: *PCCA*, *LEFTY1*, *DPEP1*, *ASCL2*, *SIVA1, MYC*, and *OLFM4*), implying a malignant intermediate stage in benign population. The marker genes also provided us with a model to investigate transition events between stages (see Fig. 4). Impressively, similar expression patterns of these genes were observed in P2 epithelial cells (Supplementary Fig. 6e). We then confirmed their distribution within tissues by projecting gene expression data back to *t*-SNE plots (Fig. 3f and Supplementary Fig. 6f).

Gene set variation analysis (GSVA) deciphered the physiological and pathological signals of the three cell types (Fig. 3g and Supplementary Fig. 6g). Signatures of nutrition absorption and metabolism were enriched in normal cell populations and were consistent with the physiological functions of intestinal epithelial cells. In benign epithelial populations, signatures of ribosome assembly and translational initiation were enriched, implying that protein synthesis is a major feature at the stage of benign adenoma. Oncogenic signaling pathways such as JAK-STAT, Wnt, and EGF were highlighted in the malignant populations. Interestingly, these cells were also enriched in some signatures related to lipid metabolisms, such as steroid biosynthesis/storage and lipoprotein clearance. These findings corroborate those of recent studies reporting that disrupted cholesterol homeostasis is involved in the carcinogenesis and progression of several cancers.^32^

To verify sample representativeness, we validated our observations with public datasets (Supplementary Fig. 7a). Similarities on enriched signatures were observed at each indicated malignant stage between our scRNA-seq data and the validation datasets (Supplementary Fig. 7b). We then investigated the expression of the marker genes using boxplot and fractional column graph. In line with the transcriptional signature, the gene expressional patterns also showed a similarity between our scRNA-seq data and the validation datasets (Supplementary Fig. 8a, b). Collectively, these results depict the molecular signatures of three canonical epithelial stages during colorectal carcinogenesis.

### Identifying precursor cell populations of adenoma and carcinoma

Identification and characterization of precursor cells help us understand the molecular mechanisms driving the initiation of adenoma or carcinoma. Considering that the evolution of CRC is a long-term continuous process, a minority population in normal, adenoma, and cancer biopsies may serve as transitional precursors during the adenoma-carcinoma sequence. Theoretically, these populations should consist of cells from two tissue origins, either normal and adenoma, or adenoma and carcinoma. Based on this hypothesis, we identified five clusters (clusters 4, 5, 6, 9, and 10) in P1 and three clusters (clusters 7, 8, and 9) in P2 with heterogeneous tissue origin (Fig. 3d and Supplementary Fig. 6d). Cluster 10 in P1 and cluster 8 in P2, which exhibited dual-signatures of immune and epithelial profiles, were excluded because these data points may come from doublet cells (Data set 1). Cluster 6 in P1, which exhibited canonical mitochondrial signatures, was excluded owing to the lack of cellular integrity. Consequently, two clusters with normal and adenoma origins (cluster 9 in P1 and cluster 9 in P2) and three clusters with adenoma and carcinoma origins (cluster 4, 5 in P1 and cluster 7 in P2) were identified as potential intermediate populations of normal-benign and benign-malignancy, respectively. Given the hypothesis that adenoma and carcinoma precursors may exist in the mixed populations of normal-adenoma and adenoma-carcinoma respectively, we further dissected and characterized the corresponding clusters.

Analysis of DEGs distinguished these mixed populations from normal, benign, and malignant epithelial cells (Fig. 3c and Supplementary Fig. 6c). We then used enrichment analysis to characterize the transcriptomic signatures of the cell populations with heterogeneous tissue origins. The mixed populations of normal-adenoma (cluster 9 in P1 and cluster 9 in P2) exhibited intensive proliferative signatures compared with the remaining clusters from physiological and benign populations (Fig. 4a and Supplementary Fig. 9a). They also did not possess a stemness signature or a basal cell profile^33^ (Fig. 4b), excluding an origin from regular stem cells. Therefore, the two clusters are probably adenoma precursor cell populations in intermediate stages between normal and adenoma cells.

Notably, compared with physiological and benign cells, the signatures of DNA replication and mismatched repair machinery were significantly enriched in the adenoma precursor cells (Fig. 4a and Supplementary Fig. 9a). The potential explanation for this result is that epithelial cells initiate DNA repair machinery to mitigate deleterious mutations that occur during the normal-adenoma transformation. To confirm the process of physiological–benign transition, we performed trajectory analysis including the sub-clusters of physiological, benign, and adenoma precursor by the Monocle algorithm. The results showed that the adenoma precursor cell population was located in the middle of pseudotime trajectories roadmap from physiological to benign stage (Fig. 4c and Supplementary Fig. 9b). Interestingly, in a trajectory analysis with a start point from normal epithelial subtypes, we observed a close connection between enterocyte type I and adenoma precursor cells in the pseudotime roadmap, implying an original evolutionary relationship (Supplementary Fig. 9c).

Next, we characterized specific marker genes to define adenoma precursor cell populations. The DEGs analysis showed that the genes specifically upregulated in these populations included *HPGDS*, *SH2D6*, *HCK*, and *BMX* (Fig. 4d and Supplementary Fig. 9d). We then evaluated the recurrence of precursor populations in our biopsy slides by probing marker proteins BMX and SH2D6 which play pivotal roles in carcinogenesis^34,35^ (Fig. 4e). Nine and fourteen of 20 patients had adenoma precursor cell population with high BMX and SH2D6 levels, respectively (Supplementary Table 3). Unexpectedly, adenoma precursor cell populations P1 and P2 had approximately 30% overlap in DEGs (Fig. 4f), implying a common pathogenic mechanism during the normal-adenoma transition. We further validated the recurrence of the adenoma precursor cell population using data from GSE166555,^36^ which consisted of patient-matched tumor and adjacent normal tissue from 12 untreated patients with CRC. We clustered adjacent normal tissue-derived epithelial cells by unsupervised graph-based clustering (Supplementary Fig. 9e). In addition to well-defined biological intestinal cell subtypes, such as enterocytes, goblet, transient amplifying, and stem cells, we also identified a population (cluster 8) sharing a large number of marker genes with adenoma precursor cell population (Supplementary Fig. 9f). We projected these cells into our *t*-SNE plot in Fig. 3a and Supplementary Fig. 6a. Remarkably, these cells merged with our identified adenoma precursor cell population, indicating an identical character of this precursor cell type (Supplementary Fig. 9g).

The carcinoma precursor cell population was more complicated than the population of adenoma precursor. Clusters 4 and 5 in P1, as well as cluster 7 in P2, comprised mixed cell populations from adenoma (21.51% in cluster 4, 27.27% in cluster 5, and 51.51% in cluster 7) and carcinoma (73.71% in cluster 4, 72.22% in cluster 5, 35.76% in cluster 7) (Fig. 3d and Supplementary Fig. 6d). Their expression signatures were similar to both benign and malignant cells (Supplementary Fig. 10a), implying the occurrence of an adenoma-carcinoma sequence. We thus denoted these cells as carcinoma precursor epithelial cells. The trajectory analysis confirmed that this population was a transition stage during the adenoma-carcinoma sequence (Supplementary Fig. 10b). Signature analysis indicated that KRAS- and p53-related pathways were significantly enriched in carcinoma precursor epithelial cells rather than in the benign and malignant populations (Supplementary Fig. 10c). We then validated the recurrence of carcinoma precursor cell population in our biopsy slides by probing their specific marker genes *SOX4* and *REG4*, which are involved in carcinogenesis^37,38^ (Supplementary Fig. 10d–f). High levels of REG4 and SOX4 were observed in seventeen and sixteen of 20 patients, respectively (Supplementary Table 3). Interestingly, clusters 4 and 5 in P1 shared very few marker genes with cluster 7 in P2 (1.11% and 2.17% overlap, respectively, Fig. 4f), implying relatively diverse pathogenic mechanisms during the adenoma-carcinoma transition. In conclusion, we identified the intermediate cell populations and characterized the signaling pathways involved in malignant transition that will contribute to improving early CRC diagnosis.

### Protein tyrosine kinases drive the normal-adenoma sequence

**Fig. 5:**
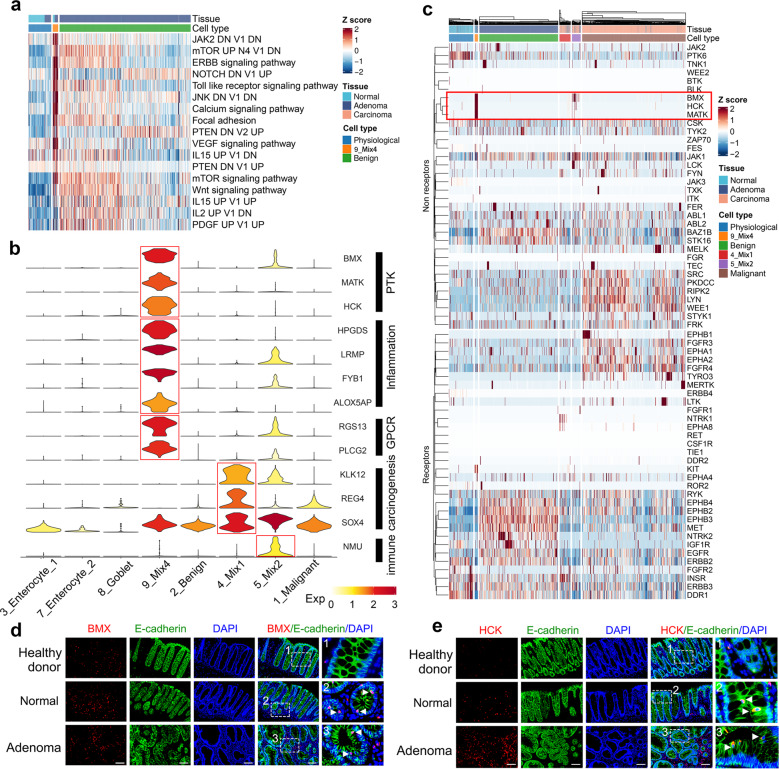
Protein tyrosine kinases drive adenoma initiation. **a** Signature scores for cells from physiological, adenoma-precursor (cluster 9_Mix4), and benign clusters. Columns represent cells annotated with cell type and tissue. Colors represent Z scores. DN, down; V1, version 1; V2, version 2. **b** Violin plot showing log-normalized expression levels of subset-specific genes in physiological (cluster 3, 7, and 8), benign, malignant, and intermediate populations (cluster 4, 5, and 9). Colors represent average expression levels within a cluster. **c** Hierarchically clustered heatmap showing the log-normalized expression levels of all expressed RTK/PTK genes in P1 epithelial cells. Columns represent cells annotated with cell type and tissue. Colors represent *Z* scores. **d**, **e** Co-immunofluorescence staining of E-cadherin with BMX (**d**) or HCK (**e**) was conducted for healthy donor colonic, CRC patient normal, and adenoma tissues. White arrows indicate positively stained epithelial cells. Scale bar, 100 μm

To identify the events driving normal-adenoma transformation, we performed gene enrichment analysis (Fig. 5a and Supplementary Fig. 11a). In line with the findings of the previous study, we found that the Wnt-β-catenin pathway was enriched in the adenoma precursor cell population, confirming the role of APC in adenoma initiation.^39^ Multiple PTK-activated pathways were also enriched in the adenoma precursor cell population. Interestingly, in adenoma precursor cells, the genes that were hit in the PTK signaling also included the marker genes of adenoma precursor cells, such as *BMX*, *MATK*, and *HCK* (Fig. 5b and Supplementary Fig. 11b). Encouraged by this evidence, we examined the roles of the PTK family in the adenoma precursor cell population by profiling their transcription. As a closely related kinase family in the kinome tree, the receptor tyrosine kinase (RTK) family members were included here as a control to validate the specificity of PTK upregulation. The results showed that *BMX*, *HCK*, and *MATK* were exclusively upregulated in the adenoma precursor cell population (Fig. 5c and Supplementary Fig. 11c). We then validated these results by probing BMX, HCK, and MATK, along with epithelial marker E-cadherin, in biopsies from healthy donors, normal tissues, and patient-matched adenoma tissues. We included tissues of healthy donors as negative controls because they lacked adenoma precursor cells. Unsurprisingly, BMX^+^ or HCK^+^ or MATK^+^ E-cadherin^+^ double-positive cells were not observed in samples from the 23 healthy donors but in both normal tissues and patient-matched adenoma tissues (Fig. 5d, e and Supplementary Fig. 12a). Quantification of fluorescence intensity of double-positive cells showed that the levels of BMX, HCK, and MATK in adenoma were higher than those in normal tissues (Supplementary Fig. 12b–d). We then evaluated the recurrence of adenoma precursor cells in a large cohort of patients with CRC. The BMX^+^ or HCK^+^ or MATK^+^ E-cadherin^+^ double-positive cells presented in both normal tissue and matched adenoma were observed in 8, 11, and 7 of 20 patients, respectively (Supplementary Table 4). Interestingly, non-epithelial cells with E-cadherin-negative showed positive staining of BMX, HCK, and MATK. The specific distribution of BMX, HCK, and MATK in minority populations cannot be detected by bulk-sequencing. Therefore, we successfully identified and characterized the adenoma precursor cell population, which might be driven by PTK signaling.

### BMX and HCK promoted epithelial cell proliferation and adenoma initiation via the JAK-STAT pathway

**Fig. 6:**
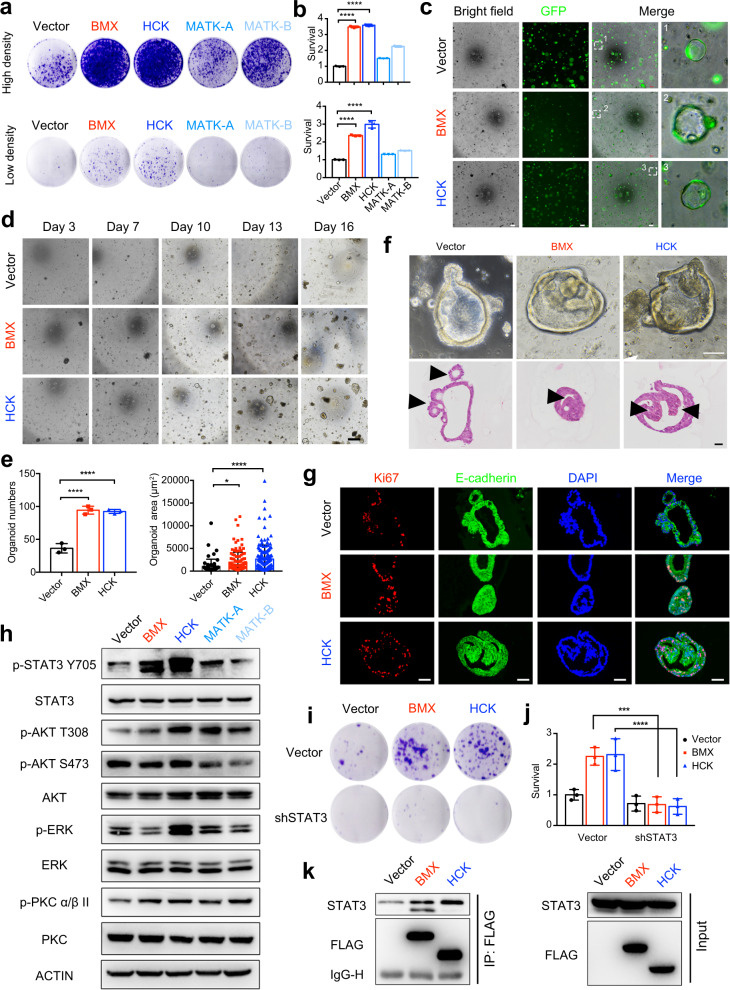
Activation of JAK-STAT pathway via BMX and HCK upregulation promotes adenoma initiation. **a** Clonogenic assays of NCM460 cells engineered to express HCK, BMX, MATK-A, MATK-B, or their vector control. **b** Quantification of **a**. **c** Representative bright-field and GFP expression images of organoids at day 5 after engineering to express BMX, HCK, or vector. Scale bar, 200 μm. **d** Time course culture of organoids expressing BMX, HCK, or vector. Scale bar, 200 μm. **e** Quantification of number and size of organoids at day 16 after engineering to express BMX, HCK, or vector. **f** Representative bright-field (up) and H&E staining (below) images of organoids engineered to express HCK, BMX, or vector. Scale bar, 100 μm. **g** Co-immunofluorescence staining of E-cadherin with Ki67 was conducted for organoids engineered to express HCK, BMX, or vector. Scale bar, 100 μm. **h** NCM460 cells were engineered to express BMX, HCK, MATK-A, MATK-B, or vector. Cell lysates were made for immunoblot analysis with indicated antibodies. ACTIN was used as a loading control. **i** Effects of STAT3 knockdown on cell proliferation in vector-, BMX-, or HCK- overexpressing NCM460 cells. **j** Quantification of **i**. **k** BMX or HCK interacted with STAT3 directly. NCM460 cells were infected with vector-, BMX-, or HCK- overexpressing plasmid. The cell lysates were immunoprecipitated with an anti-FLAG antibody, then the precipitates and cell lysates were analyzed using western blotting with the indicated antibodies. *P < 0.05; **P < 0.01; ***P < 0.001; ****P < 0.0001; analyzed using ANOVA

To validate the biofunction of the PTKs, we overexpressed BMX, HCK, and MATK-A/B in the NCM460 cell line, an immortalized normal intestinal epithelial cell line lacking intrinsic expression of these genes^40^ (Supplementary Fig. 13a, b). A long-term proliferation assay showed that BMX and HCK, but not MATK-A and MATK-B, significantly promoted cell proliferation, indicating that BMX and HCK could induce hyper-proliferative characteristics in normal colorectal epithelial cells (Fig. 6a, b). A 3-day MTT assay indicated that the overexpression of BMX and HCK improved short-term cell proliferation (Supplementary Fig. 13c). Interestingly, overexpression of BMX and HCK increased the number of 3D-clonal spheroid of NCM460 cells (Supplementary Fig. 13d, e). To test whether this hyper-proliferation causes polyp formation, we infected the human intestinal organoids (an ex vivo model) with lentivirus carrying GFP-tagged BMX or HCK open reading frames (Fig. 6c). BMX or HCK upregulation significantly increased organoid proliferation in terms of number and size (Fig. 6d, e). Notably, we observed multiple polyp-like buds protruding towards the lumen and hyperplasia of the wall in BMX- or HCK-upregulated organoids (Fig. 6f). Small buds protruding outward from the basal layer are signs of organoid growth and differentiation,^41–44^ which were also observed in our control organoids. In contrast, the polyp-like structure protruding toward the lumen mimics polyp morphology in clinics. Cells in this polyp-like structure lost contact inhibition, leading to the formation of a multi-layered organoid. Consistently, Ki67 staining followed by quantification indicated that the cell proliferation rates in organoids with overexpression of BMX and HCK were higher than those in the control organoids (Fig. 6g and Supplementary Fig. 13f).

To elucidate the molecular mechanism, we dissected the signaling pathways that were potentially driven by BMX and HCK. According to the GSVA analysis, the PTK-related signaling pathways were enriched, including calcium, AKT/mTOR, JAK-STAT, etc. (Fig. 5a and Supplementary Fig. 11a). We found that the phosphorylation level of BMX or HCK was upregulated in NCM460 cells upon the overexpression of BMX or HCK respectively, implying activation status (Supplementary Fig. 13a). We then probed the phosphorylation levels of STAT3, AKT, ERK, PKC, FOXO3a, PDK1, β-catenin, and GSK3β, which are involved in the downstream pathways of PTK. The results showed that STAT3 was hyper-phosphorylated in response to ectopic BMX and HCK expression (Fig. 6h). Interestingly, after introducing all four PTKs, a slight upregulation of AKT and PKC phosphorylation was observed in MATK-A/B overexpressed cells. Considering that MATK-A/B did not promote cell proliferation or adenoma formation, we speculate that AKT or PKC activation may not directly mediate adenoma initiation. Moreover, we detected the phosphorylation levels of FOXO3a, PDK1, β-catenin, and GSK3β, which are related to the AKT pathways. None of them was activated upon the overexpression of these PTKs (Supplementary Fig. 13g). To verify the function of STAT3 in BMX or HCK promoted epithelial cell proliferation, we knocked down STAT3 in NCM460 cells with the overexpression of BMX or HCK (Supplementary Fig. 13h). The results showed that cell proliferation promoted by BMX or HCK overexpression was reversed by STAT3 knockdown (Fig. 6i, j). In addition, direct interactions between STAT3 and BMX or HCK were validated using co-immunoprecipitation (CoIP) assay (Fig. 6k). Taken together, our results indicate that BMX and HCK promote colorectal epithelial cell proliferation and adenoma formation through the STAT3 signaling pathway.

## Discussion

In the past decades, the driver events and molecular mechanisms underlying the colorectal adenoma-carcinoma sequence have been intensively investigated using bulk sequencing.^31^ However, prior to this study, the evolution of epithelial cells and associated molecular signatures at a single-cell transcriptome level remained unexplored. Here, we simultaneously collected a series of patient-matched samples and performed scRNA-seq to trace CRC evolution. We systematically characterized the dynamic evolution of distinct epithelial cell populations from normal tissue to adenoma and finally to carcinoma.

Epithelial cell populations were defined as three typical stages (physiological, benign, and malignant) using GSVA and DEGs analyses, which were validated by public databases. During successive stages of tumorigenesis, the precursor cells may comprise a minority population with intermediate features.^45^ For example, the adenoma precursor cell population may include mixed populations of normal plus adenoma cells. Likewise, carcinoma precursors may consist of adenoma plus carcinoma cells. Based on this hypothesis, we successfully identified these two intermediate populations using transcriptional signatures and trajectory analyses. Furthermore, we found that BMX or HCK potentially drove normal-adenoma transition, whereas *KRAS* activation and *p53* dysregulation drove adenoma-carcinoma transformation. Notably, BMX or HCK alone drove the formation of polyp-like structures in organoid models, emphasizing their strong influence on adenoma initiation. We then confirmed the existence and distribution of these precursor cells via immunohistochemistry (IHC) evaluation of marker genes in a large cohort. These observations fill the gaps in the evolutionary roadmap of colorectal carcinogenesis and may improve our understanding of mechanisms underlying malignant transformation in CRC. Our results may facilitate the development of prevention strategies for populations susceptible to CRC.

In conclusion, this study focused on the evolution of epithelial cells during colorectal carcinogenesis. Taking advantage of patient-matched scRNA-seq data, we characterized epithelial cell populations at each stage of the adenoma-carcinoma sequence and identified potential precursor cells initiating adenoma and carcinoma. Our datasets will be valuable as a resource for further exploration of the molecular mechanisms underlying malignant transformation during colorectal carcinogenesis. Furthermore, our research provides a theoretical basis for early prevention and the development of novel therapeutic regimens targeting the malignant transformation of epithelial cells for CRC.

## Materials and methods

### CRC patients

This study was approved by the local ethics committee of West China Hospital, Sichuan University, and with written informed consent collected. Only patients with untreated, non-metastatic CRCs that underwent radical resection were included in this study.

### Preparation of single-cell suspensions

Fresh peripheral blood was collected prior to surgery in an EDTA anticoagulant tube and subsequently layered onto the ice. Following resection in the operating room, indicated samples were obtained immediately according to the designed collecting criterion of each tissue. Samples from cancer and polyp tissues were collected from the outer upper quadrant when placed the resected specimen in a longitudinal direction. Samples from the normal tissues were at least 5 cm from the matched cancer tissues, while samples from the adjacent normal tissues were <2 cm from the matched cancer tissues. All samples were divided into segments, a part of them were placed in liquid nitrogen or neutral buffered formalin for processing to formalin-fixed paraffin-embedded blocks, and others were collected for generating single-cell suspension. Samples for single-cell suspension were preserved in DMEM (Hyclone) supplemented with 10% fetal bovine serum (NATOCOR), 1% penicillin and streptomycin (Millipore), and transported rapidly to the research facility. On arrival, each sample was subsequently minced on ice to smaller pieces of <1 mm^3^ and transferred to 15 ml digestion medium containing 0.25% trypsin (Millipore). Samples were incubated for 15 min at 37 °C, with manual shaking every 2 min. Samples were then pipetted up and down for 1 min using pipettes of Pasteur pipette (BIOFIL). Next, 20 ml ice-cold DMEM, containing 10% fetal bovine serum was added and samples were filtered using a 70-μm strainer (BIOFIL). Following centrifugation using a swing-out rotor at 400 × *g* and 4 °C for 5 min, the supernatant was decanted and discarded, and the cell pellet was resuspended in 2 ml red blood cell lysis buffer and transferred to a 2 ml DNA low bind tube. Following a 5-min incubation at room temperature, samples were centrifuged (500 g, 4 °C, 5 min). Samples were next resuspended in 500 μl PBS containing 0.04% BSA (Sigma) and filtered over 70-μm cell strainers (BIOFIL).

The prepared cells were sorted by flow cytometry to isolate indicated cell populations with high bioactivities. Briefly, the cells were stained with propidium iodide to exclude dead cells. Sorted cells were then counted and assessed for viability with Trypan blue using a Countess II automated counter. Cells were then resuspended at a 6 × 10^5^–1.2 × 10^6^ cells/ml concentration with final viability of >80% as determined with the Countess.

### Droplet-based scRNA-seq

Single-cell library preparation was carried out according to the protocol of the 10x Genomics Chromium single-cell v2 reagent kit (10x Genomics, Pleasanton, CA, USA). Libraries were sequenced on an Illumina HiSeq 4000 at a depth of 50,000–100,000 reads/cell.

### Exome sequencing

Genomic DNA of patient-matched tissue samples was extracted using the QIAamp genomic DNA kits (QIAGEN) according to the manufacturer’s specification. DNA concentrations were quantified using NanoDrop. Next, sonication was used to fragment DNA, and DNA was sheared to 150–200 bp in length. Then the library was prepared with SureSelectXT Human All Exon v6 kits (Agilent Technologies) and sequenced on an Illumina novaSeq 6000.

### Exome analysis

Reads were mapped to the reference human genome (GRCh38) using BWA v.0.7.17. Picard v.2.20.0 was used to mark duplicate reads and GATK v.4.1.2.0 to realign. We applied MuTect to detect somatic nucleotide variation and INDEL by comparing the sequencing reads of cancer and matched normal genomes. Annotation was performed with SnpEff v.4.3t. Copy number profiles were carried out by sequenza v.3.0.0 software. The variant allele frequencies were filtered with copy number profiles. Initial clusters were identified using PyClone v.0.13.0 by shared mutation in different cancer samples. The PyClone cluster frequencies were calculated as the mean variant allele frequencies of mutations within each cluster. The clonal frequencies were then adjusted using citup v.0.1.2^46^ by joint calculation of the cluster identifications and optimal trees across the tumor time points from the same patient. Microsatellite instability in carcinoma samples was called using MSIsensor v.0.5.

### Pre-processing scRNA-seq data

We aligned to the GRCh38 reference genomes as appropriate for the input dataset, and estimated cell-containing partitions and associated unique molecular identifiers (UMIs), using the Cell Ranger v.3.0.0 Single-Cell Software Suite from 10X Genomics, and 54,788 cells were identified totally. The scimpute R package v.0.0.9 was used to correct for drop out using the following parameter settings k-means spectral clustering (*k* = 15), but otherwise, default parameters. Then Seurat R package v.3.10^29^ was used to pre-process single-cell data. Genes expressed in fewer than three cells in a sample were excluded, as were cells that expressed fewer than 200 genes or mitochondrial gene content >25% of the total UMI count. We normalized data with the default normalize method that normalizes the gene expression measurements for each cell by the total expression, multiplies this by a scale factor (10,000), and log-transforms the result.

### Dimensionality reduction, clustering, and differential expression analysis

We performed unsupervised clustering and differential gene expression analyses in Seurat. First, we identified the top 2000 highly variable features by the FindVariableFeatures function, focusing on these genes was helpful to highlight biological signals in downstream analysis. Next, we applied a linear transformation to scale the expression of each gene, so that the mean is 0 and the variance is 1. Then we perform PCA, a linear dimensional reduction technique on the scaled data, only the top 2000 highly variable features were used as input. We remained 40 components for the merged object and 20 components for the individual objects (P1 epithelial and P2 epithelial) according to the Elbow plot. To cluster these cells, we first applied FindNeighbors function to construct a K-nearest neighbor graph according to the Euclidean distance in PCA space, and edge weights between two cells were refined by the shared overlap in their local neighborhoods. Previously defined dimensionalities were used as input. We next apply the Louvain algorithm to iterate group cells together. This step was performed through FindClusters function, and the resolution parameter was set at 1 to merged objects and 0.6 to individual objects (P1 epithelial and P2 epithelial). Then, we conducted differential gene expression analysis using the standard AUC classifier to assess significance. We contrasted cells from that sub-cluster to all other cells of that sub-cluster using the FindMarkers function. To evaluate the robustness of clustering, we optimized the clustering by integrating the scclusteval^47^ algorithm (with default parameter) and biological annotation. According to these DEGs of each sub-cluster and canonical cell markers, 19 biologically meaningful cell types were annotated.

### Batch effect correction

Firstly, we clustered cells with the raw count. The results showed that the normal epithelial cells (enterocytes and goblet cells) from different patients merged well, while the epithelial cells from adenoma and carcinoma were distributed discretely, which reflects the biological differences between tissues. Then, we use harmony (V1.0) to remove batch effects from patients with default parameters. These results suggested minimal batch effects of our scRNA-seq data and no need to remove them.

### Sub-clustering of the major cell types

To study the evolution of cells at an individual level, we performed re-clustering on P1 epithelial and P2 epithelial, respectively. The procedure of re-clustering is the same as previous, starting from unfiltered expression matrix, including finding high variable genes, scaling data, performing dimensional reduction, and clustering cells.

### Gene set variant analysis

Pathway analyses were performed on C2.CP.KEGG, C5.BP, C6, and C7 gene sets in Molecular Signatures Database (MSigDB v6.2^48^) by using GSVA R package v1.34.0^49^ with default parameter. Then limma R package v3.42.0^50^ was used to find differential gene sets, we contrasted cells from that sub-cluster to all other cells of that sub-cluster. The threshold of differential gene sets was set to *p* < 0.05. The enrichment of each pathway was indicated by the normalized enrichment score.

### Single-cell signature scores

SingleCellSignatureExplorer v3.6 was used to compute single-cell signature scores of P1 and P2 epithelial cells. This software computes in each cell a score for any gene set. C2.CP.KEGG, C5.BP, C6, and C7 gene sets of MSigDB v7.0 were used here. The score of gene set *GSx* in the cell *Cj* was computed as the sum of all UMI for all the *GSx* genes expressed in *Cj*, divided by the sum of all UMI expressed by *Cj*. As gene numbers in each gene set are highly variable, a single-cell score for a signature cannot be compared to that of other signatures.

### Pseudotime analysis

The single-cell trajectories were constructed by Monocle v2.14.0 R package.^51^ Monocle learns the sequence of gene expression changes each cell must go through as part of a dynamic biological process, and constructs a trajectory that mainly reflects the progress of cells moving from the starting state. We created a CellDataSet object for single-cell of each cell type with the default parameter. Two major steps were then performed for single-cell trajectory construction. The first step was to choose genes that could provide important information in defining the progress. Ordering genes were isolated by comparing the cells at the beginning state of the process to those at the end and finding the DEGs, and exon sequencing results have demonstrated the sequential relationship between normal and adenoma and carcinoma. The second step was dimensionality reduction and trajectory construction with the ordering genes. Reversed graph embedding algorithm was applied in this process, by projecting cells to a low dimensional space while simultaneously learning smooth tree-like manifold.

### Cell culture

NCM460 was cultured in RPMI-1640 medium supplemented with 10% fetal bovine serum and 1% penicillin/streptomycin (100 IU/ml) in a humidified incubator at 37 °C with 5% CO_2_.

### Immunoblotting

NCM460 cells were washed twice with pre-cold PBS before lysis in RIPA buffer (Millipore) supplemented with phosphatase inhibitor and protease inhibitor (Bimake). The BCA Protein Assay Kit (Thermo) was used to determine the protein concentrations.

Cell lysates or immunoprecipitates were separated by 10% SDS-PAGE gels and then transferred to PVDF membranes (Millipore). After being blocked with 5% BSA for 1 h, the members were incubated with primary and secondary antibodies.

### Co-immunoprecipitation

CoIP was performed as previously reported.^52^ In brief, cell lysates were incubated with FLAG antibody at 4 °C for 4 h, then protein G-agarose beads were added into cell lysates at 4 °C overnight. Beads were then washed with PBS with 0.1% Tween 20 and subjected to western blot analysis.

### Clonogenic assay

NCM460 cells were plated in six-well plate and allowed to adhere overnight. On the following day, BMX, HCK, MATK, and CPPT viral supernatants were added to the plates in the presence of 6 μg/ml polybrene for 24 h. For the research of STAT3 knockdown, the cells were then infected with shRNA or vector control (pLL3.7) viral supernatants. After 24 h of incubation at 37 °C under 5% CO_2_, the medium was replaced with fresh RPMI complete medium. After infection, the medium was changed every 2 days. Colonies were fixed in 4% paraformaldehyde and stained with 0.5% crystal violet after two weeks. The crystal violet was dissolved in 0.1 M sodium Citrate in 50% acetic acid and measured OD at 590 nm.

### Organoid culture

Crypts were isolated as described previously.^41,42^ Briefly, human normal intestinal fragments were washed with cold DPBS, then incubated in 5 mM EDTA with gentle shaking at 4 °C for 30–40 min. The isolated healthy crypts were counted and embedded in Matrigel (Corning, #356237) and cultured in IntestiCult^TM^ Organoid Growth Medium (StemCell Technology, # 06010) or Human Intestinal Stem Cell medium (HISC, comprised with advanced DMEM/F12 medium, GlutaMAX, HEPES, penicillin, streptomycin, N2, B27, *N*-acetylcysteine, noggin, R-spondin 1, EGF, WNT3a, A83–01, SB202190, FGF10, nicotinamide, gastrin, Prostaglandin E2, and Y27632). The medium was changed every 2 or 3 days.

### Lentiviral cDNA/shRNA constructs and retrovirus production

shRNA and cDNAs were cloned into the pLL3.7 and pRRLsin.cPPT.CMV.GFP (denoted as CPPT) lentiviral vectors, respectively. Recombinant retroviruses were generated by third-generation lentiviral packaging using human embryonic kidney (HEK) 293T cells as previously described.^53^ The sequence of shRNA targeting STAT3 used was as follows: STAT3-shRNA: GAGATTGACCAGCAGTATA.

### Cell proliferation assay

Cell proliferation was monitored by MTS (Promega, #G3582) following the manufacturer’s recommendations. NCM460 cells infected with vector, BMX, or HCK virus were seeded in 96-well plates for 24 h. Then the cell proliferation was monitored by MTS (Promega, #G3582) at the indicated time following the manufacturer’s recommendations.

### Spheroid formation assay

Cells were embedded in Matrigel (BD, 356234) in 96-well plate and cultured at 37 °C for 2 weeks. Medium replenished every 2 days. Pictures of spheroids were taken with the microscope (Nikon). The number of spheroids was counted at the low power objective (×4). The experiment was performed in triplicate.

### Transduction of crypts

For infections of crypts, they were disassociated into single cells by TrypLE (Gibco) and washed with DPBS. After centrifuging, crypts were diluted in 500 μl growth medium and 500 μl infectious viral supernatant (concentrated with Lenti-X Concentrator (Takara)) with polybrene at the final concentration of 6 μg/ml were added to 12-well plates. To promote contact between the crypts and viral particles, plates were centrifuged after adding the cells, 1000 × *g* for 1 h. After centrifuging, the cells were incubated in a CO_2_ incubator overnight. The following day, the crypts were embedded in Matrigel.

### Immunohistochemistry and immunofluorescence staining

To prepare the samples for IHC staining, the tissues were fixed with 10% formalin followed by paraffin embedding. Tissue sections of 4 µm thickness were mounted on glass slides for hematoxylin and eosin (H&E), IHC, or immunofluorescence staining. The slides were deparaffinized, incubated in 3% hydrogen peroxide, antigen retrieval was performed in EDTA for 3 min in a pressure cooker. The slides were incubated with individual primary antibodies at 4 °C overnight, followed by incubation with appropriate HRP-conjugated secondary antibodies for IHC or Alexa 488-, and Alexa 647-conjugated secondary antibodies specific to the species of the primary antibodies with DAPI for immunofluorescence staining. Antibody were used including anti-SH2D6 (Abcam, #ab185810, 1:200), anti-BMX (Santa Cruz, #sc-376686, 1:50), anti-HCK (Promab, #20166, 1:200), anti-SOX4 (Abcam, #ab80261, 1:200), anti-REG4 (Proteintech, #11268-1-AP, 1:100), anti-Ki-67 (CST, #9027 s, 1:200), and anti-E-cadherin (CST, #3195 s, 1:100). IHC staining was scored according to the following standards: staining percentage was designated as: − (<10%), + (<25%), ++ (25–50%), +++ (51–75%), or ++++ (>75%).

## Supplementary information

supplementary figures and tables

Dataset 1

## Data Availability

Data sets generated in this study using scRNA-seq have been deposited at the GEO database under accession code “GSE161277”. All data generated or analyzed during this study are included in this published article and its supplementary information files. All data in this study are available from the corresponding author with a reasonable request.
