# Supplementary material for: Single-cell transcriptomic profiling unravels the adenoma-initiation role of protein tyrosine kinases during colorectal tumorigenesis
Source: Signal Transduct Target Ther. 2022 Feb 28;7:60. doi: 10.1038/s41392-022-00881-8 (PMC8882672; doi:10.1038/s41392-022-00881-8)
Supplement: Supplementary file 1 — supplementary figures and tables [file 41392_2022_881_MOESM1_ESM.docx]

Supplementary Materials for

Single-cell Transcriptomic Profiling Unravels the Adenoma-initiation Role of Protein Tyrosine Kinases in Colorectal Tumorigenesis

Xiaobo Zheng^1, 2†^, Jinen Song^1†^, Chune Yu^1†^, Zongguang Zhou^3†^, Xiaowei Liu^1^, Jing Yu^1^, Guangchao Xu^1^, Jiqiao Yang^1^, Xiujing He^1^, Xin Bai^1^, Ya Luo^1^, Yu Bao^1^, Huifang Li^4^, Lie Yang^5^, Mingqing Xu^6^, Nan Song^7^, Xiaodong Su^8^, Jie Xu^9^, Xuelei Ma^10*^, Hubing Shi^1, 2*^

Correspondence to: [shihb@scu.edu.cn](mailto:shihb@scu.edu.cn); drmaxuelei@gmail.com

**This PDF file includes:**

Figures. S1 to S13

Tables S1 to S4

Captions for Data S1

**Other Supplementary Materials for this manuscript include the following:**

Data S1. DEGs of epithelial subtypes in P1 and P2.

**
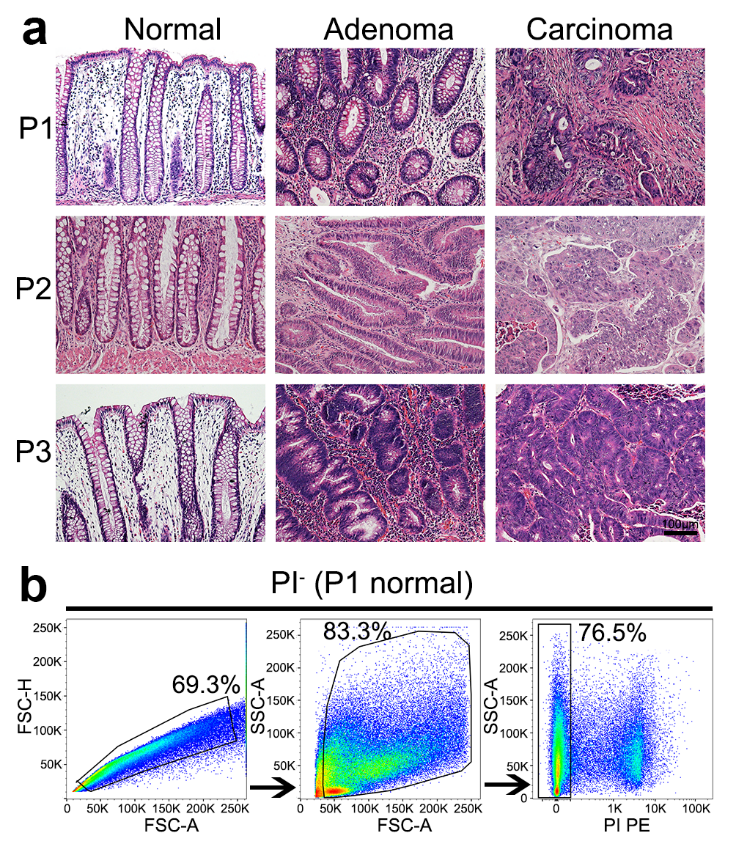
**

**Figure. S1. Pathological confirmation and FACS sorting.**

**(a)** Collected samples were pathologically confirmed as normal tissue, adenoma, or carcinoma using H&E staining. Scale bar, 100 μm. **(b)** Gating strategy for single-cell sorting in this study (exemplified by P1 normal tissue). All cell types were enriched by sorting for PI^-^.


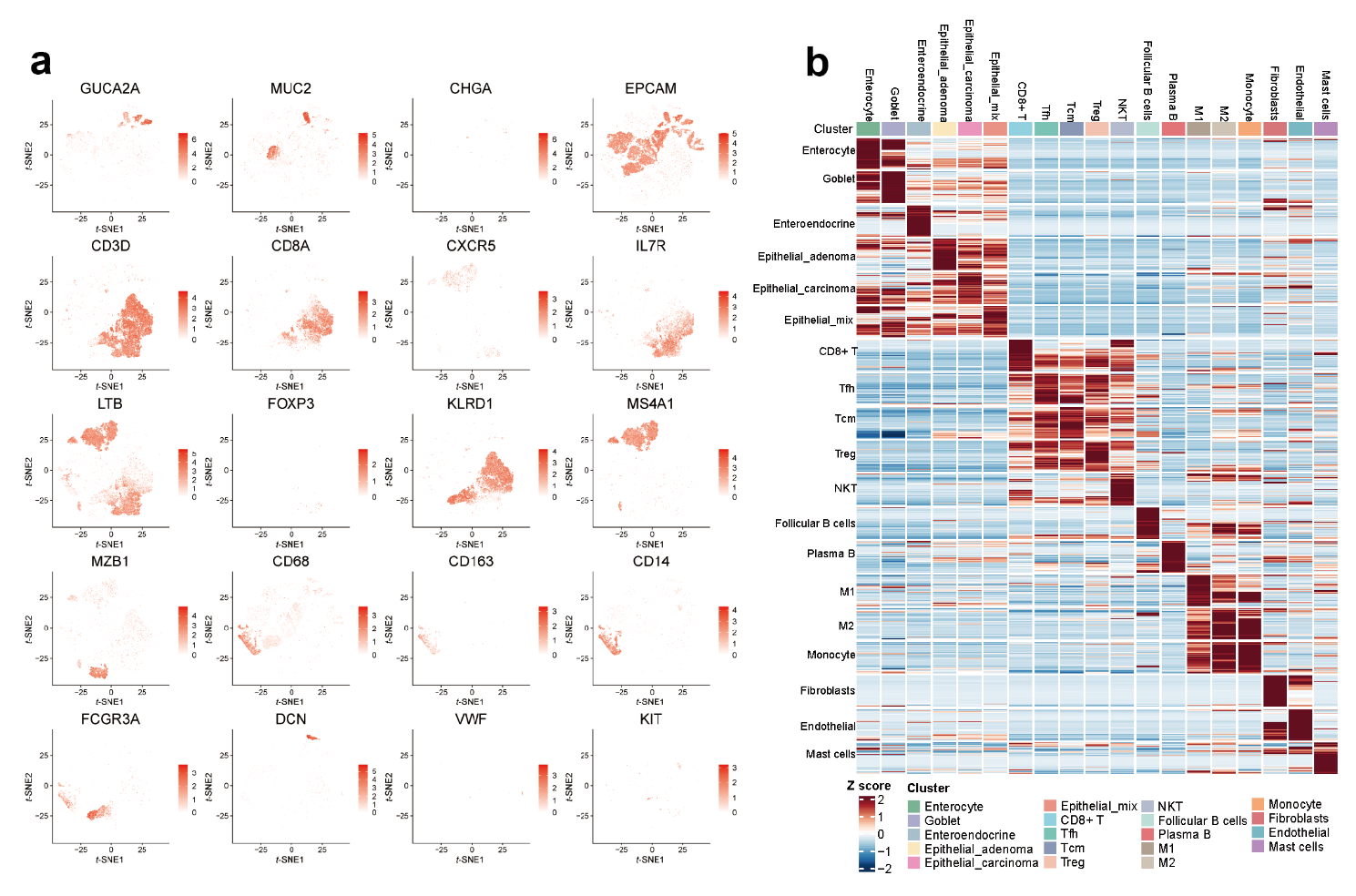


**Figure. S2. Robustness of cell typing and clustering.**

**(a)** Expression of marker genes in enterocyte (*GUCA2A*), goblet (*MUC2*), enteroendocrine (*CHGA*), epithelial (*EPCAM*), T (*CD3D*), CD8+ T (*CD8A*), follicular helper T (*CXCR5*), central memory T (*IL7R*), regulatory T (*FOXP3*), natural killer T (*KLRD1, CD8A*), follicular B (*MS4A1*), plasma B (*MZB1*), M1 macrophage (*CD68*), M2 macrophage (*CD163*), monocyte (*CD14, FCGR3A*), fibroblasts (*DCN*), endothelial (*VWF*), and mast (*KIT*) cells. Colors indicate the log-normalized data, and each dot represents a single cell. **(b)** Heatmap of the top 50 differentially expressed genes of 19 sub-clusters. Columns represent cell types. Rows indicate genes, and the colors represent the Z-Score of log-normalized data.


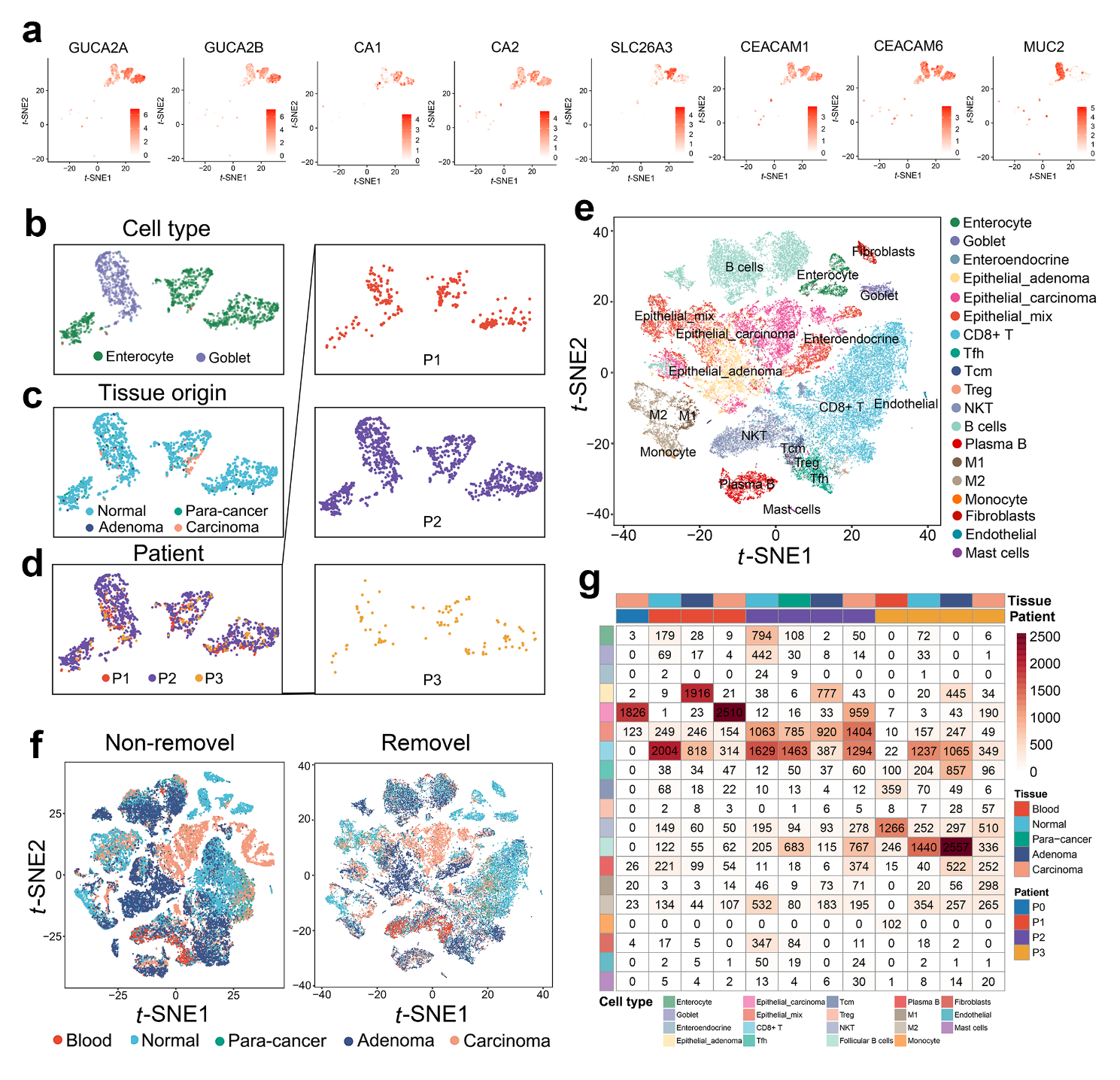


**Figure. S3. Batch effect exclusion.**

**(a)** Expression of key marker genes in enterocytes and goblet cells (*GUCA2A, GUCA2B, CA1, CA2, SLC26A3, CEACAM1, CEACAM6, MUC2*). Colors indicate the log-normalized data, and each dot represents a single cell. **(b)** *t*-SNE plot of 1,869 enterocytes and goblet cells. Each dot represents a single cell. **(c)** *t*-SNE plot of enterocytes and goblet cells colored by tissue origin. **(d)** *t*-SNE plot of enterocytes and goblet cells and the three faceted *t*-SNE plots colored by patient origin. **(e)** *t*-SNE plots of cells from four patients (12 samples) after removing the batch effect using harmony. Colors represent cell types. **(f)** *t*-SNE plots showing the difference in tissue distribution before (left) and after (right) removing the batch effect. Colors correspond to cell types. **(g)** Heatmap showing the cell numbers of each cell type in 12 samples, with rows representing cell types and columns representing samples.


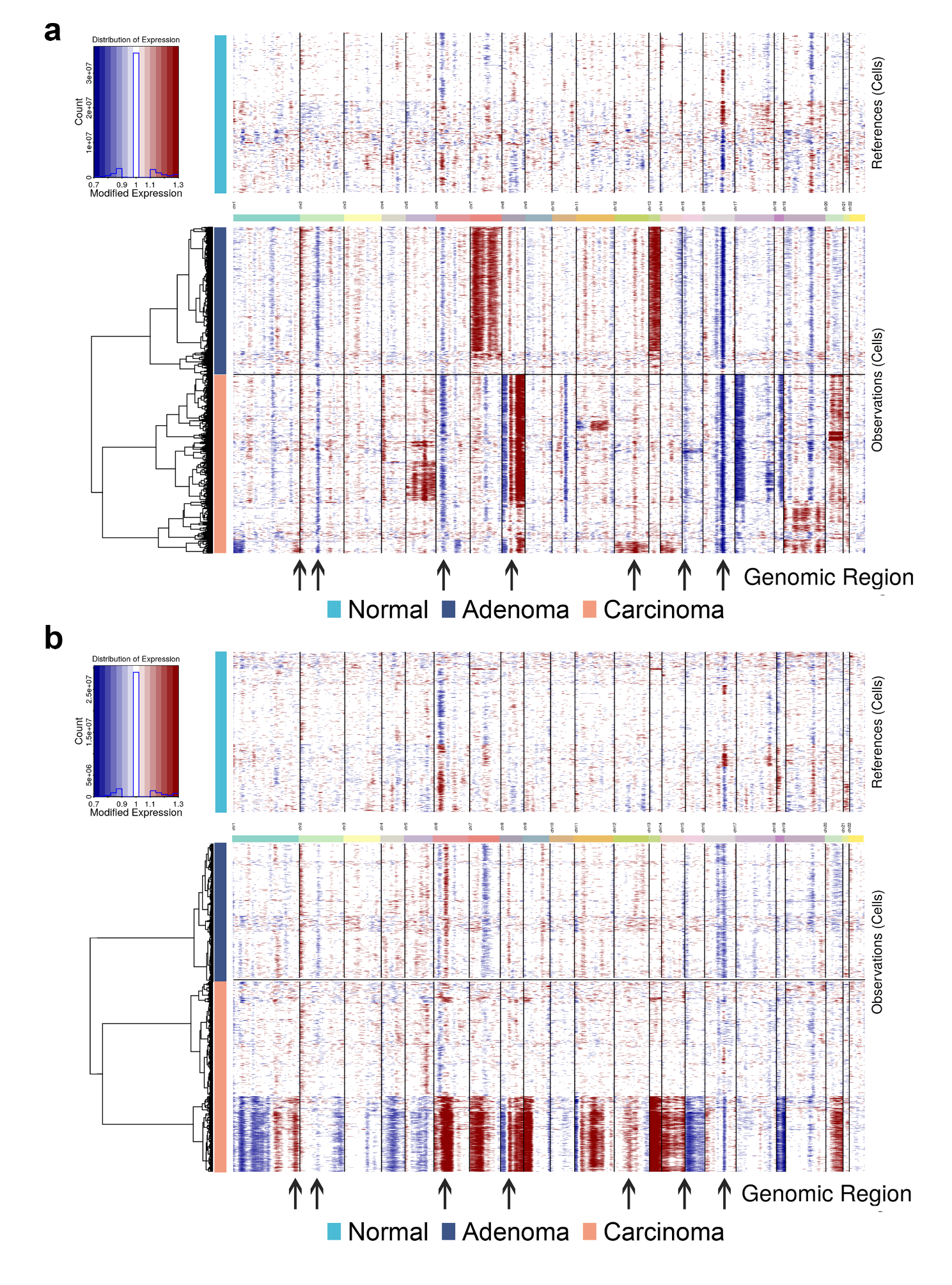


**Figure. S4. Inference of copy number variation from scRNA data.**

**(a-b)** Heatmap showing the inferCNV result on P1 (**a**) and P2 (**b**) epithelial cells. Normal tissue origin epithelial was used as a reference. Colors represent inferCNV scores. Each row indicates a single cell, and each column represents a position in the chromosome. Common CNVs between adenoma and carcinoma are marked with arrows.

**
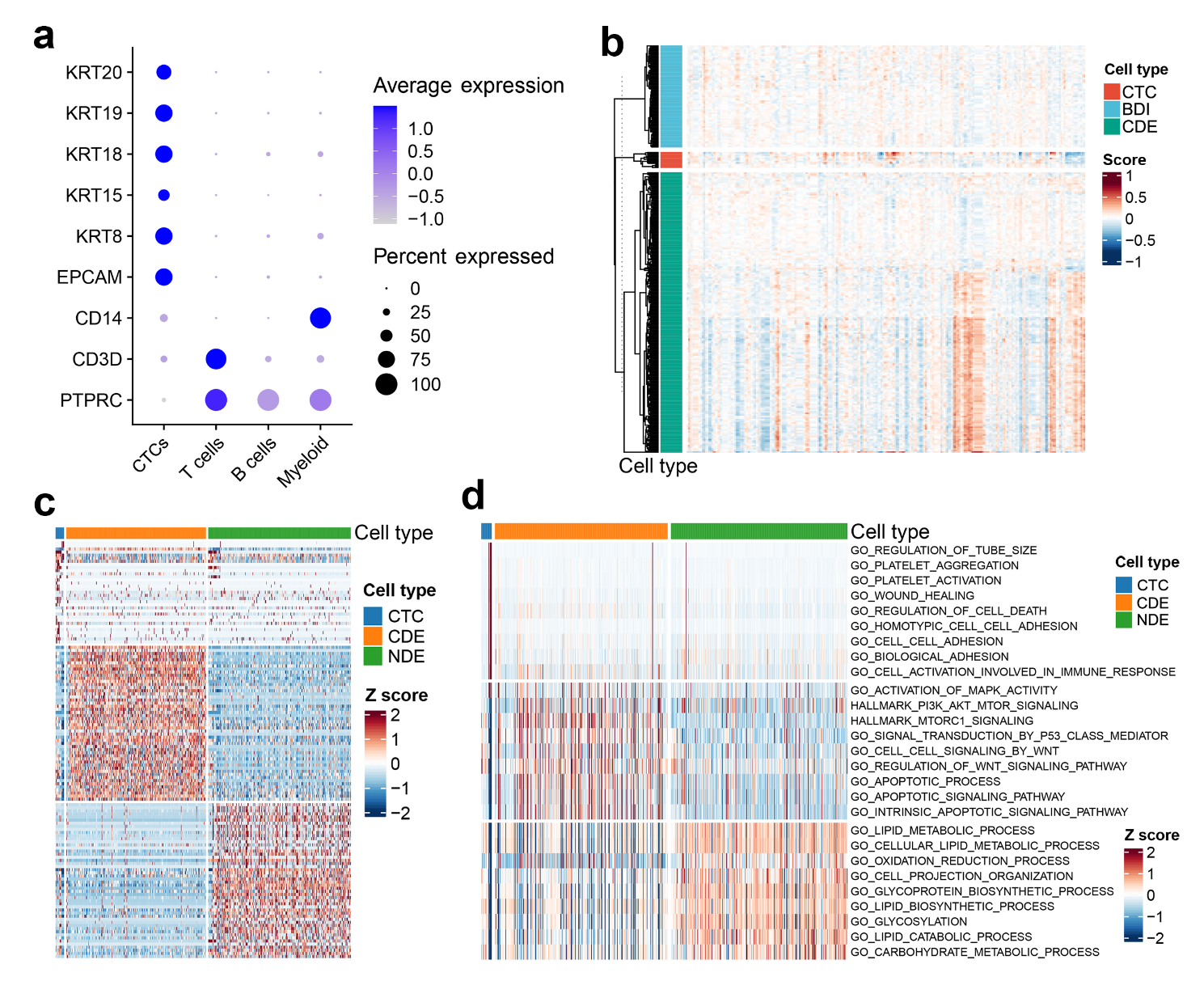
**

**Figure. S5. Circulating tumor cells identified in the blood of P3.**

**(a)** Dot plot showing the expression level of immune and epithelial genes. Columns indicate cell sub-cluster. Circle size represents the percentage of cells that express the gene, and colors represent the average expression of log-normalized value within a cluster. **(b)** Heatmap showing the inferCNV result of CTC, BDI, and CDE. BDI was used as a reference. Colors represent inferCNV scores. Rows correspond to cells, and columns indicate the position in the chromosome. **(c)** Heatmap showing the top 50 differentially expressed marker genes of each cell type. Columns denote individual cells annotated with cell type. Rows indicate genes, and colors represent Z-Score of log-normalized data. **(d)** GSVA results of CTC, CDE, and NDE. Rows represent gene sets, columns represent cells, and colors indicate GSVA scores for each cell. CTC, circulating tumor cells; BDI, blood-derived immunocytes; CDE, cancer-derived epithelial cells; NDE, normal-derived epithelial cells.

**
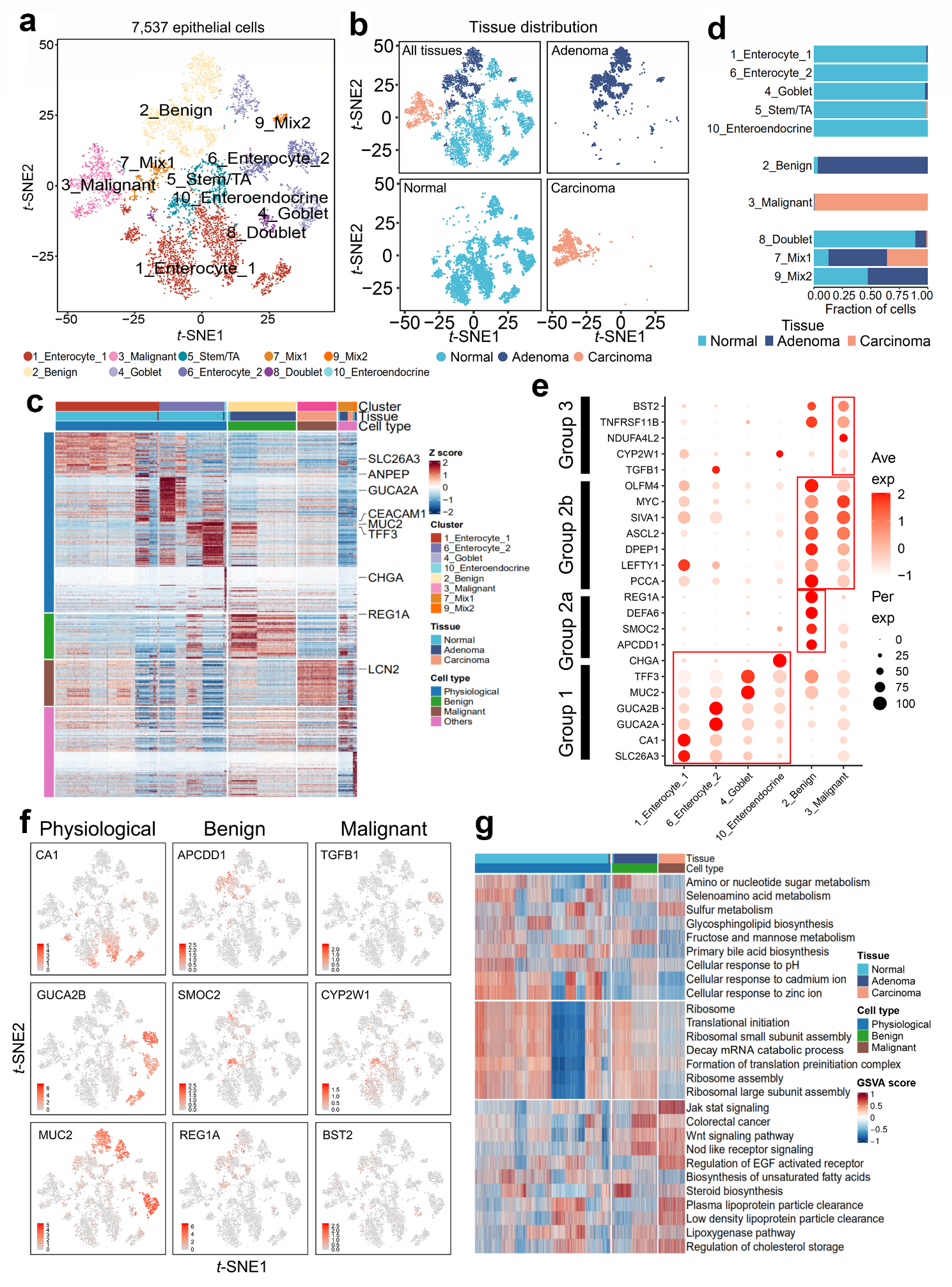
**

**Figure. S6. Validation of three typical stages of epithelial cells during colorectal carcinogenesis in P2.**

**(a)** *t*-SNE plot of 7,537 epithelial cells in P2. These cells were defined as 10 cell sub-clusters based on biological annotation and inferCNV analysis. Each dot represents a single cell, and colors correspond to cell types. **(b)** *t*-SNE plot of P2 epithelial cells and three faceted *t*-SNE plots colored by tissue origin. Non-malignant epithelial cells of carcinoma origin were grouped into normal origin. **(c)** Heatmap of the top 50 differentially expressed marker genes of each cluster. Columns denote individual cells annotated with cell type, tissue, and cluster. Normal epithelial cell clusters (1_Enterocyte_1, 6_Enterocyte_2, 4_goblet, and 10_Enterodocine) were set as physiological. Rows indicate genes annotated with cell type (left) and exemplar gene names (right). Colors represent Z-Score of log-normalized data. **(d)** Fraction of tissue in each cell sub-cluster. **(e)** Dot plot of physiological, benign, and malignant clusters by the expression of cluster-specific genes. Columns indicate cell sub-cluster. Circle size represents the percentage of cells that express the gene, and the colors represent the average expression of log-normalized value within a cluster. **(f)** The log-normalized expression level of marker genes in physiological cells (*CA1*, *GUCA2B*, *MUC2*), benign cells (*APCDD1*, *SMOC2*, *REG1A*), and malignant cells (*TGFB1*, *CYP2W1*, *BST2*). **(g)** GSVA results in physiological, benign, and malignant cells. Rows represent gene sets, and columns represent cells annotated with cell type and tissue. Colors indicate GSVA scores for each cell.

**
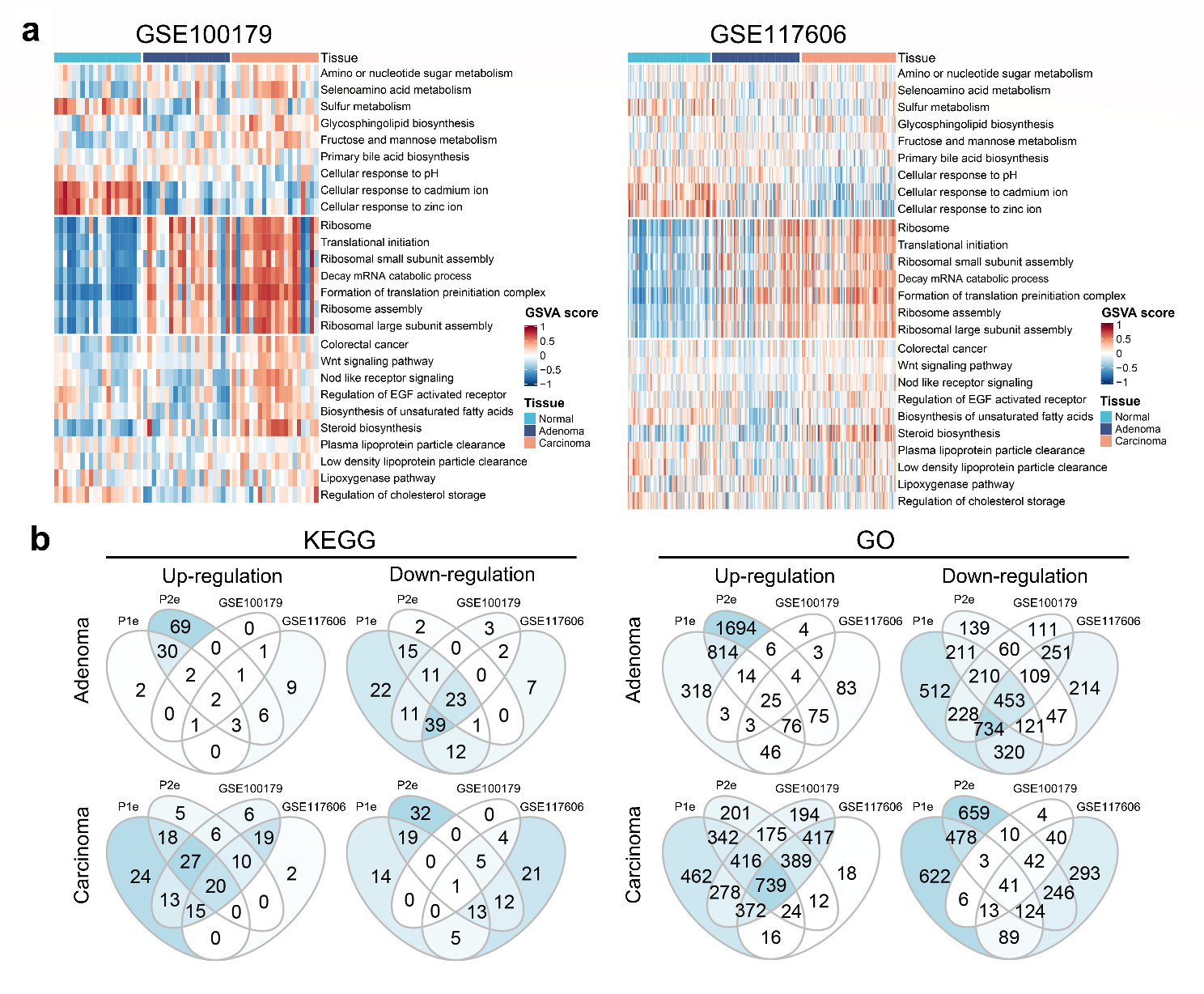
**

**Figure. S7. Validation of gene sets representativeness of our samples with public datasets.**

**(a)** GSVA results of normal, adenoma, and carcinoma tissues. Gene expression matrix of GSE100197 (60 samples) and GSE117606 (208 samples) were downloaded from the GEO database. Rows represent gene sets and columns represent cells annotated with tissue. Colors indicate GSVA scores. **(b)** Upregulated and downregulated gene sets of KEGG (left) and GO (right) in adenoma and carcinoma intersected across datasets of P1 epithelial (P1e), P2 epithelial (P2e), GSE100179, and GSE117606.


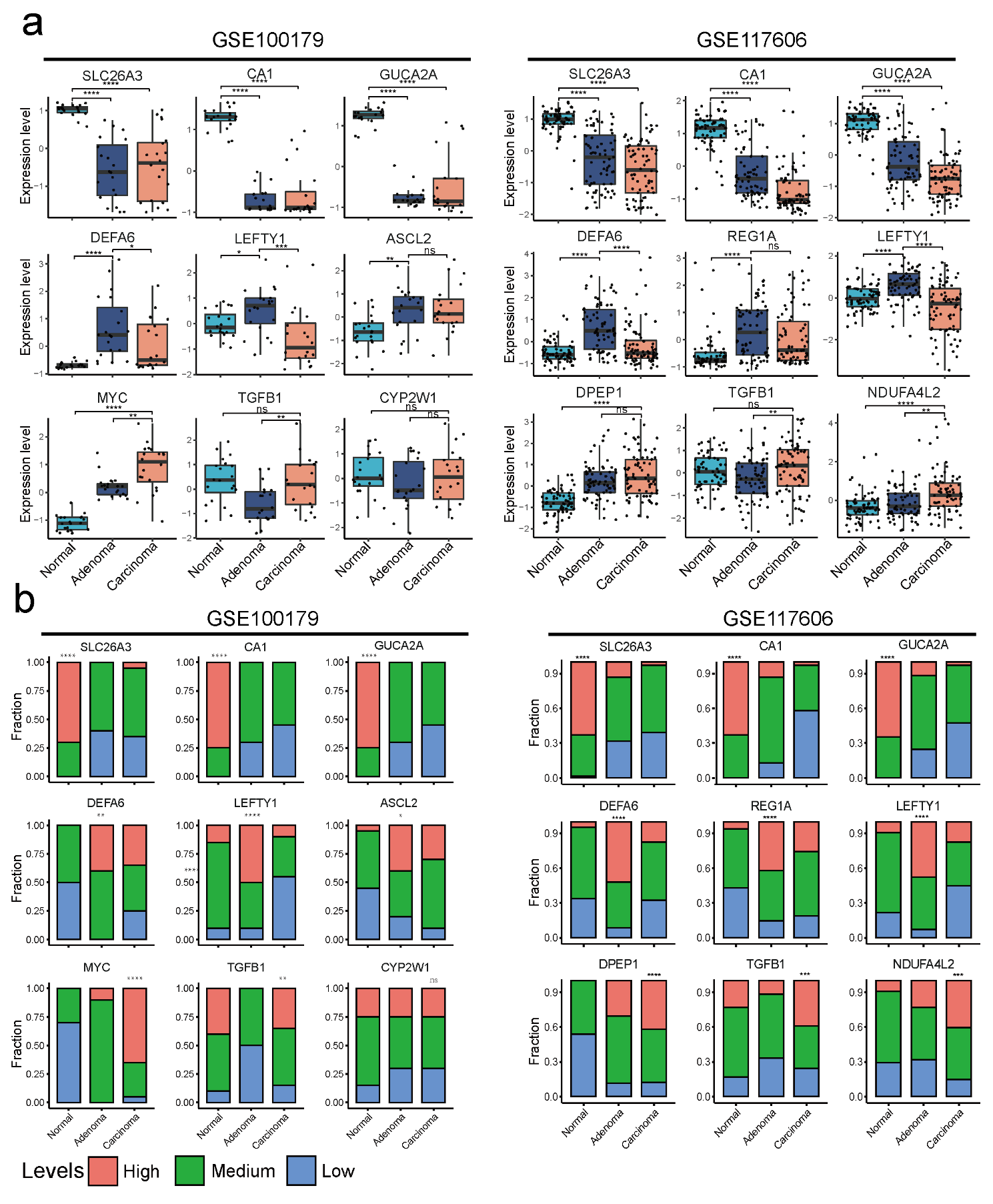


**Figure. S8. Validation of genes representativeness of our samples with public datasets.**

**(a)** Representative differentially expressed genes among normal, adenoma, and carcinoma tissues were validated with datasets GSE100197 (left) and GSE117606 (right). Statistical significance was tested by the two-sided Wilcox test. **(b)** Column graph showing the representative differentially expressed genes among normal, adenoma, and carcinoma tissues in validation datasets. The gene levels in the top 25%, 25%-75%, and bottom 25% were defined as high, medium, and low expression, respectively. Statistical significance was tested by the chi-square test. ns, no significance; *, *p* < 0.05; **, *p* < 0.01; ***, *p* < 0.001; ****, *p* < 0.0001.


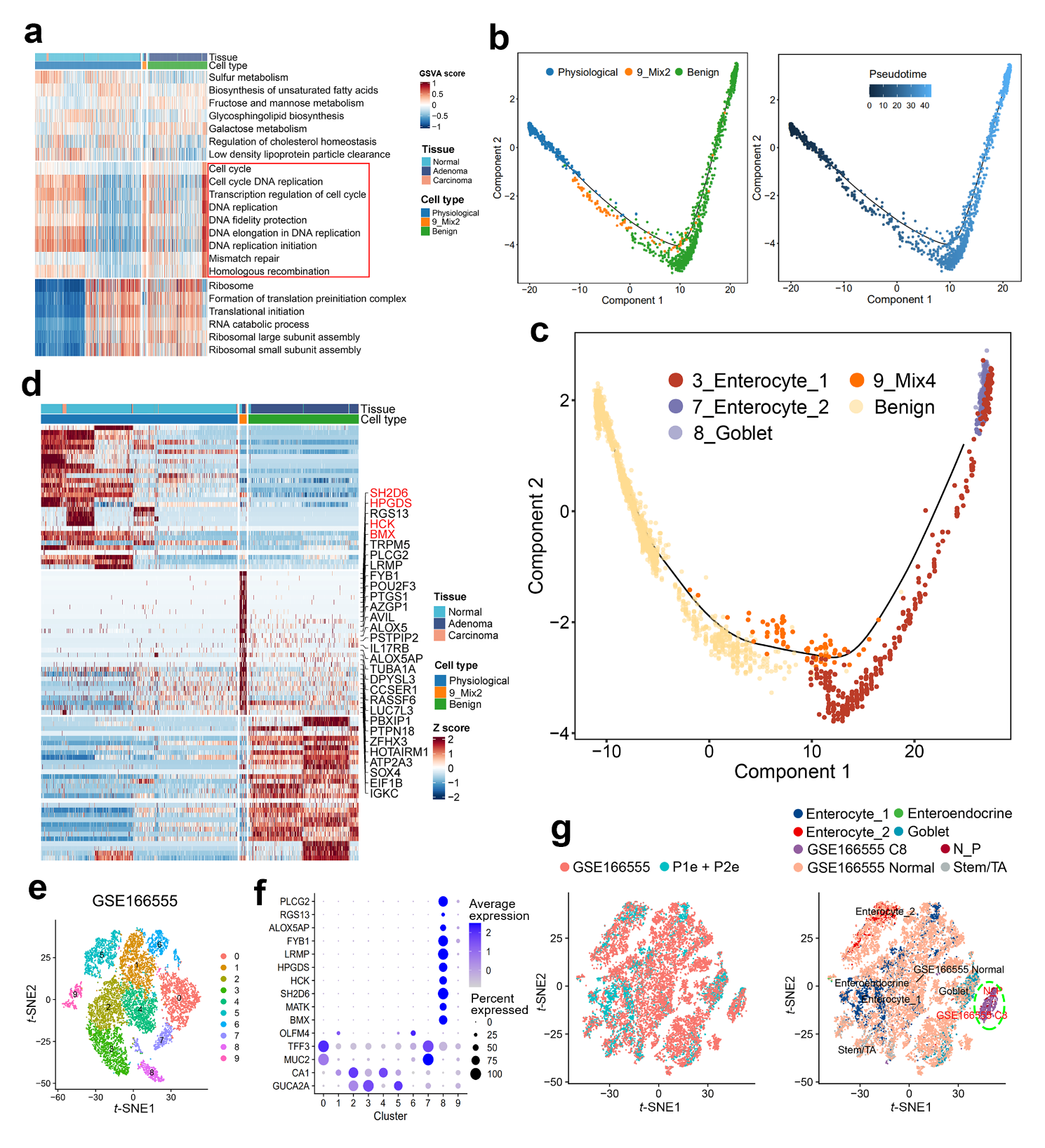


**Figure. S9. Identified normal-adenoma intermediate populations in P2 and public scRNA dataset.**

**(a)** Signature scores of physiological, benign, and mixed cluster (cluster 9) in P2. Columns represent cells annotated with cell type and tissue. Colors indicate Z scores. **(b)** Single-cell trajectory of physiological, adenoma-precursor (cluster 9), and benign epithelial cells. The trajectory was constructed by monocle according to gene expression. Each dot represents a single cell. Colors represent cell types (left) and pseudotime of the trajectory (right). **(c)** Single-cell trajectory of 3_enterocyte_1, 7_enterocyte_2, 8_goblet, adenoma-precursor cells (cluster 9), and benign epithelial cells. Each dot represents a single cell, and colors represent cell types. **(d)** Heatmap of differentially expressed genes among normal, adenoma precursor (cluster 9), and adenoma epithelial cells. Genes related to carcinogenesis were highlighted. **(e)** All epithelial cells from normal tissues in dataset GSE166555 were clustered into 10 sub-clusters. **(f)** Dot plot showing the expression level of normal intestinal cells and adenoma precursor cells mark genes. Circle size represents the percentage of cells that express this gene, and the colors represent the average expression of log-normalized value within a cluster. **(g)** Normal epithelial and adenoma precursor cells in P1 and P2 were integrated with all normal tissues from the validation dataset GSE166555. The batch effect was corrected by canonical correlation analysis (left). GSE166555 cluster 8 merged with our identified adenoma precursor cell population (N_P, right).


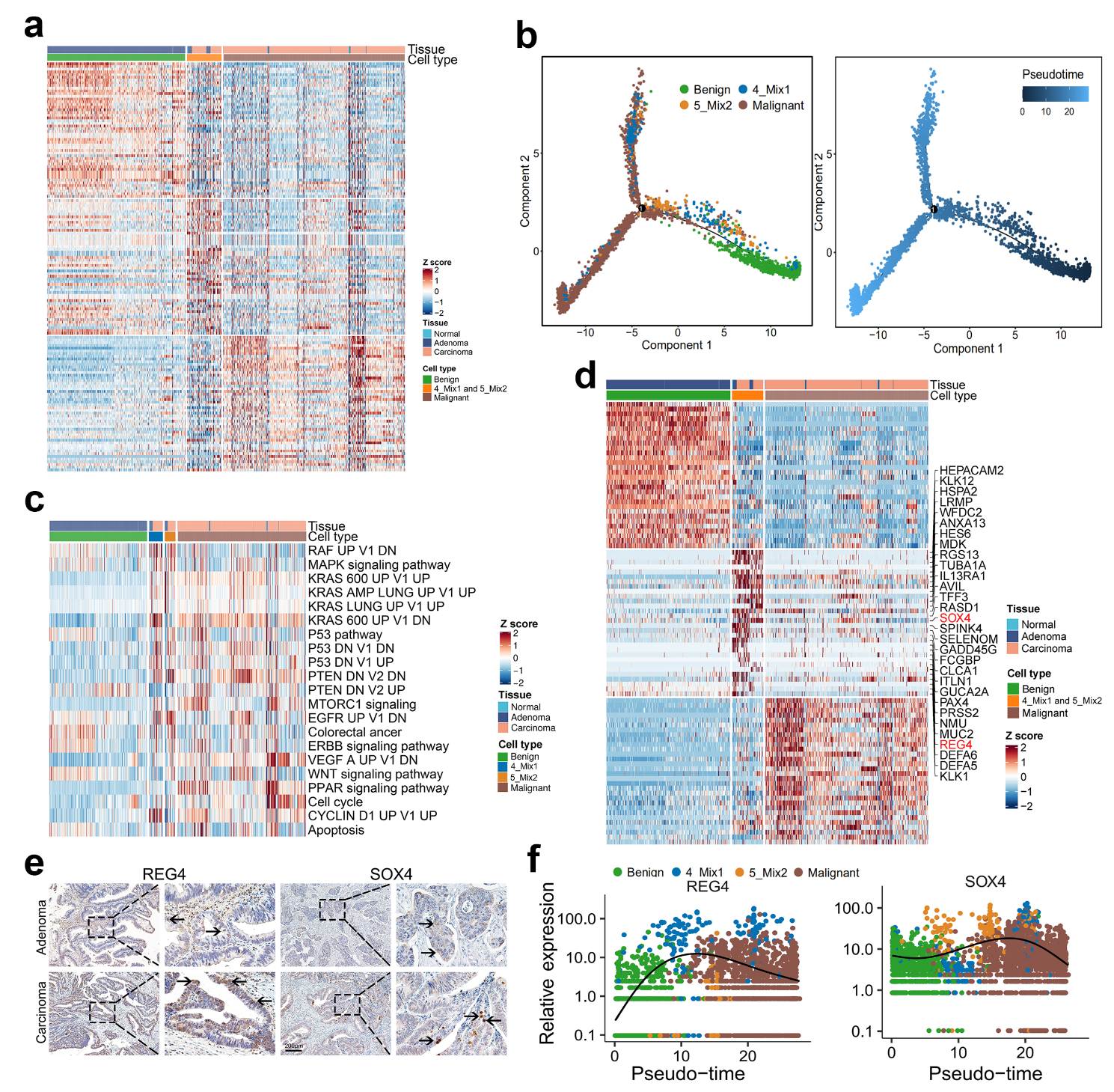


Figure. S10. Identified intermediate populations between adenoma and carcinoma in P1.

**(a)** Top 50 differentially enriched pathways of each cell type. Columns represent cells annotated with cell type and tissue. Colors represent Z scores. **(b)** Single-cell trajectory of 1,793 benign, 449 carcinoma-precursor (cluster 4 and 5), and 2,360 malignant epithelial cells. The trajectory was constructed by monocle according to gene expression. Each dot represents a single cell. Colors represent cell types (left) and pseudotime (right). **(c)** Signature scores of cells (n = 4,602) from benign, carcinoma-precursor, and malignant clusters. Columns were annotated as (a). Colors represent Z scores. **(d)** Heatmap of differentially expressed genes among adenoma, carcinoma precursor, and carcinoma epithelial cells. Genes related to carcinogenesis were highlighted. **(e)** Representative images of REG4 and SOX4 IHC staining of adenoma (n = 20) and carcinoma (n = 19). Black arrows indicate positively stained cells. Scale bar, 200 μm. **(f)** Dot plot showing the dynamic expression changes of REG4 (left) and SOX4 (right) with pseudotime. X-axis represents pseudotime, Y-axis represents gene expression level, and colors correspond to cell type.

**
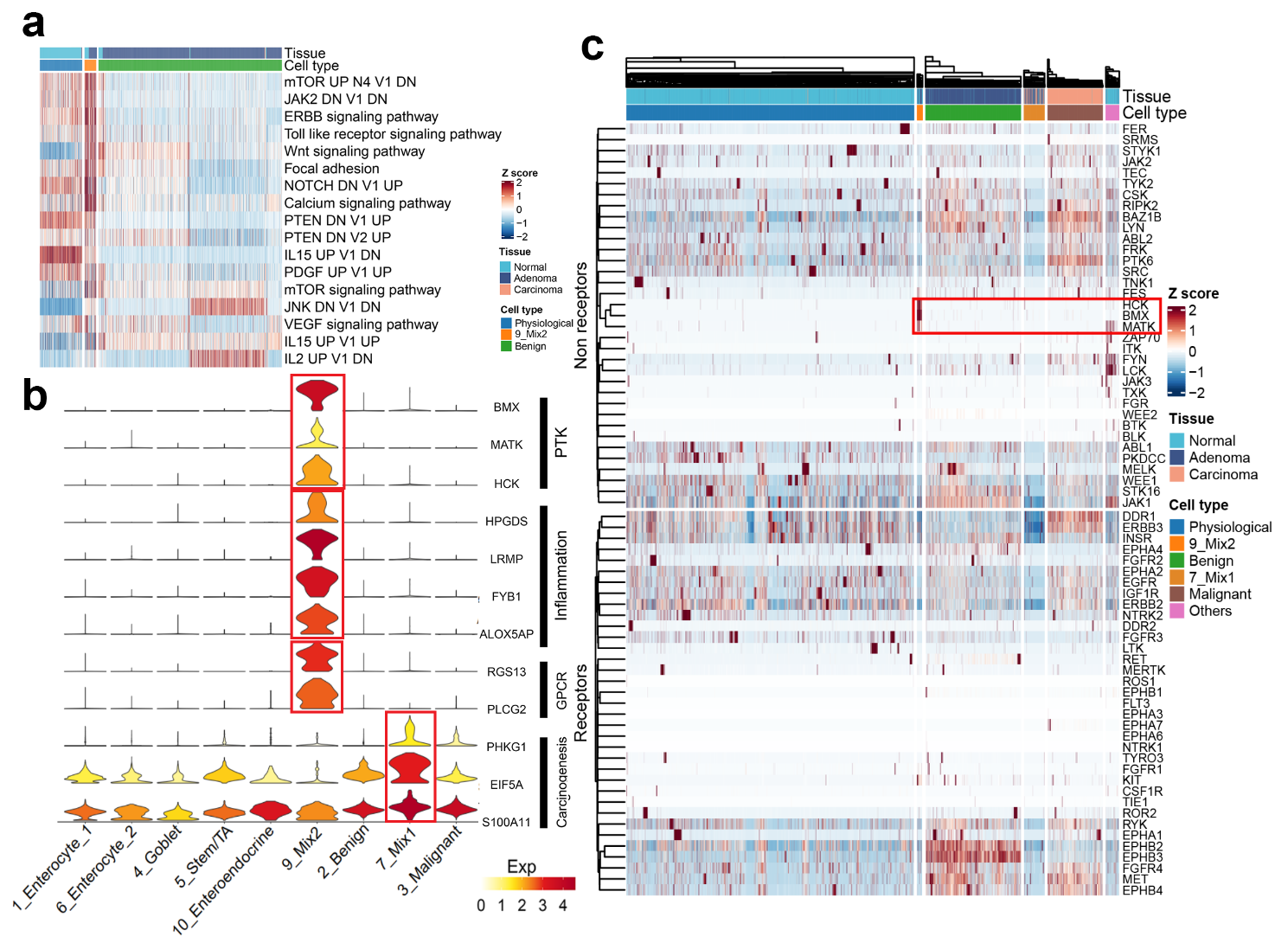
**

**Figure. S11. Validation of the driving role of protein tyrosine kinases in adenoma initiation.**

**(a)** Signature scores of cells from physiological, adenoma-precursor (cluster 9), and benign clusters. Columns represent cells annotated with cell type and tissue. Colors represent Z scores. DN, down; V1, version 1; V2, version 2. **(b)** Violin plot showing log-normalized expression levels of subset-specific genes in physiological, benign, malignant, and intermediate populations (cluster 9 and 7). Colors represent average expression levels within a cluster. **(c)** Hierarchically clustered heatmap showing the log-normalized expression levels of all expressed RTK/PTK genes in P2 epithelial cells. Columns were annotated as (a). Colors represent Z scores.

**
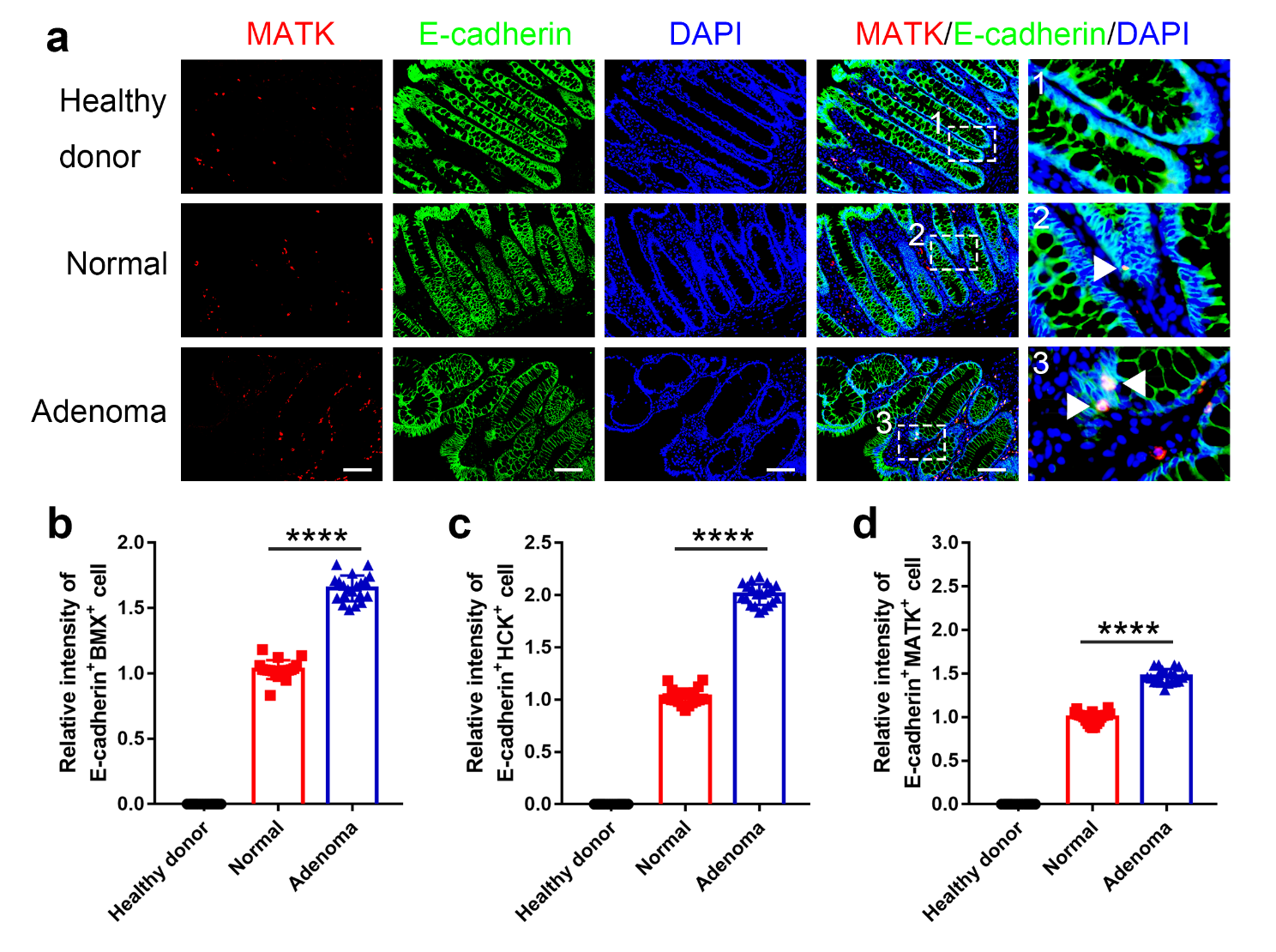
**

**Figure. S12. Expression of BMX, HCK, and MATK were upregulated in adenoma precursor cells.**

**(a)** Co-immunofluorescence staining of E-cadherin with MATK were conducted for healthy donor colonic, CRC patient-matched normal and adenoma tissues. White arrows indicate positively stained epithelial cells. Scale bar, 100 μm. **(b-d)** Quantification of the relative intensity of positive cells in Figure 5d (**b**), e (**c**) and figure S12a (**d**). ****, *P*<0.0001.

**
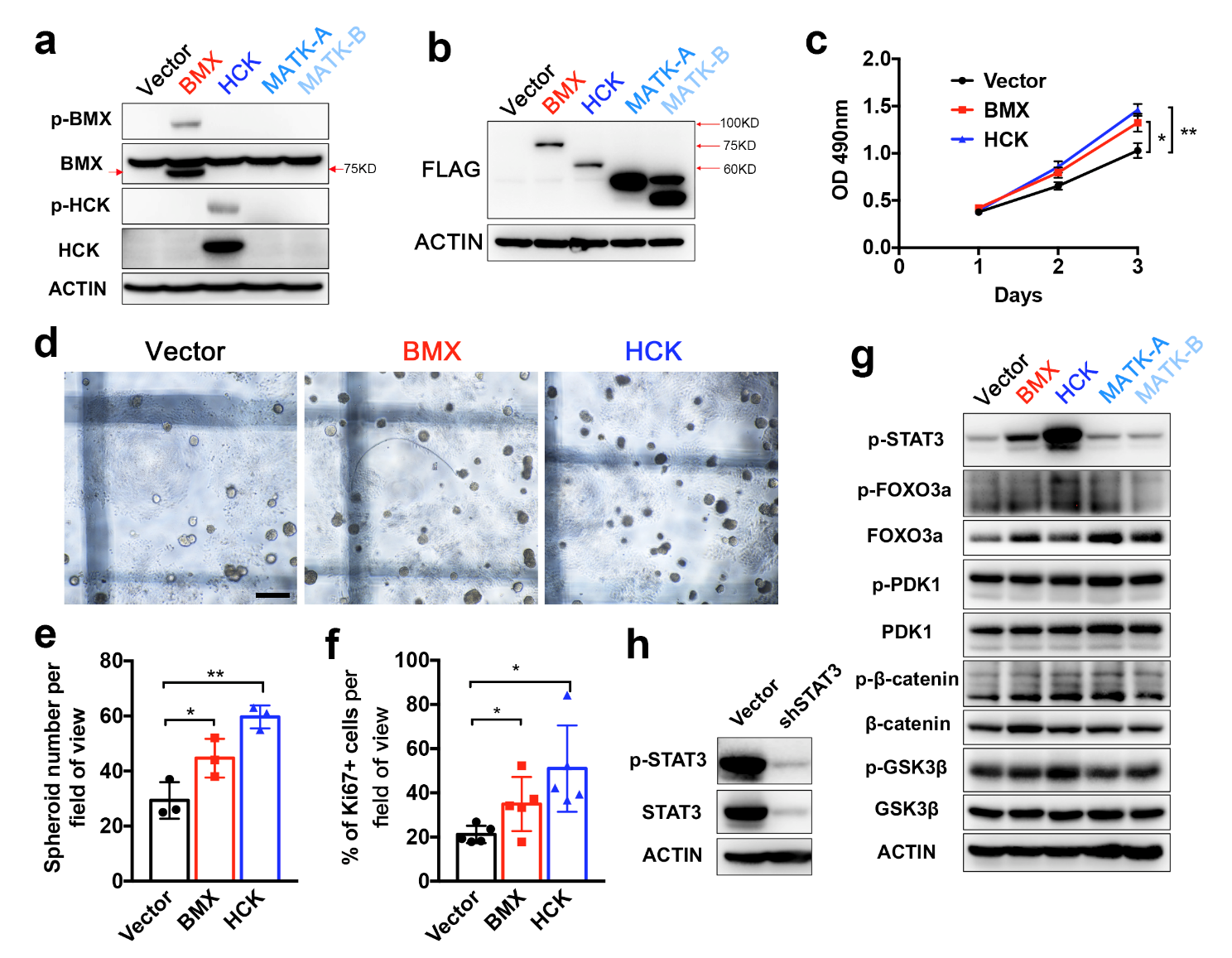
**

**Figure. S13. BMX or HCK promoted adenoma initiation through regulating STAT3.**

**(a)** Western blot analysis of indicated proteins in NCM460 cells infected with vector, BMX, HCK, MATK-A, or MATK-B virus. ACTIN was used as a loading control. **(b)** Western blot analysis of FLAG expression in NCM460 cells infected with vector, FLAG-tagged BMX, HCK, MATK-A, or MATK-B virus. ACTIN was used as a loading control. **(c)** MTT assays of vector, BMX, or HCK overexpressed NCM460 cells. **(d)** Representative phase-contrast images of NCM460 cells overexpressed vector, BMX, or HCK in a 3D culture system. Scale bar, 400 μm. **(e)** Quantification of spheroid number in **(d)**. **(f)** Quantification of Ki67 positive cells in Figure 6g. **(g)** Western blot analysis of NCM460 overexpressed vector, BMX, HCK, MATK-A, or MATK-B. **(h)** Western blot confirmed the efficiency of shRNA targeting STAT3. *, *P*<0.05; **, *P*<0.01.

| **Table S1. Clinical characteristics of four CRC patients in this study.** | | | |  |
| --- | --- | --- | --- | --- |
| **Patient ID** | **Patient 0 (P0)** | **Patient 1 (P1)** | **Patient 2 (P2)** | **Patient 3 (P3)** |
| **Age** | 46 | 61 | 64 | 53 |
| **Gender** | Female | Male | Male | Female |
| **Site of tumor** | Colon | Colon | Rectum | Colon |
| **Histological type of tumor** | ADC^a^ | ADC | ADC | ADC |
| **Histological type of polyp** | none | tubulovillous adenoma | tubulovillous adenoma | tubulovillous adenoma |
| **tissue processed** | cancer | normal/polyp/cancer | normal/para-cancer/polyp/cancer | blood/normal/polyp/cancer |
| **pTNM: T** | 3 | 3 | 4a | 3 |
| **pTNM: N** | 0 | 1 | 0 | 0 |
| **pTNM: M** | 0 | 0 | 0 | 0 |
| **pTNM** | pT3pN0 | pT3pN1b | pT4apN0 | pT3pN0 |
| **Stage** | IIA | IIIB | IIB | IIA |
| **Tumor size** | 4.3×3.8×1.7 cm | 6.1×2.3×0.4 cm | 5.5×2.5×1.3 cm | 4.5×3.5 ×1.2cm |
| **Grade** | G2~G3 | G2~G3 | G2 | G2~G3 |
| **MSI status^b^** | NA^c^ | MSS | MSS | MSS |
| **Genotype** | NA | APC、KRAS、PIK3CA | TP53 | APC、KRAS、FAT4 |
| **IHC** | MLH1 (+), MSH2 (+) | MLH1 (+), MSH2 (+) | MLH1 (+), MSH2 (+) | MLH1 (+), MSH2 (+) |
|  | MSH6 (+), PMS2 (+) | MSH6 (+), PMS2 (+) | MSH6 (+), PMS2 (+) | MSH6 (+), PMS2 (+) |
|  | Ki67 (+), CDX2 (+) | Ki67 (+), CDX2 (+) | Ki67 (+), CDX2 (+) | Ki67 (+) |
|  |  | Desmin (+) | CK20 (+) |  |
| ^a^ ADC, adenocarcinoma. ^b^ MSS, microsatellite stability; MSI, microsatellite instability. ^c^ NA, not available. | | | | |

| **Table S2. Characteristics of the twelve tissue samples included in this study.** | | | | | | | | | |
| --- | --- | --- | --- | --- | --- | --- | --- | --- | --- |
| **patient** | **tissue type** | **data size（G)** | **total reads** | **mean reads per cell** | **median UMI counts per cell** | **number of cells** | **mean genes per cell** | **fraction reads per cell** | **saturation** |
| 0 | carcinoma | 89 | 318,155,621 | 107959 | 6177 | 2947 | 1753 | 94.00% | 87.80% |
| 1 | normal | 90 | 342,999,587 | 88676 | 3710 | 3868 | 1362 | 92.50% | 85.60% |
| 1 | adenoma | 90 | 355,033,810 | 86215 | 8900 | 4118 | 2233 | 95.70% | 76.00% |
| 1 | carcinoma | 120 | 495,741,947 | 128364 | 14143 | 3862 | 3452 | 94.00% | 73.80% |
| 2 | normal | 159 | 580,107,911 | 76845 | 3569 | 7549 | 1191 | 93.60% | 84.60% |
| 2 | para-cancer | 109 | 407,429,236 | 84669 | 3344 | 4812 | 1154 | 92.20% | 85.60% |
| 2 | adenoma | 89 | 356,516,660 | 81340 | 5056 | 4383 | 1382 | 91.20% | 76.60% |
| 2 | carcinoma | 139 | 569,843,284 | 76468 | 3497 | 7452 | 1191 | 94.00% | 78.40% |
| 3 | blood | 90 | 359,601,743 | 164577 | 2663 | 2185 | 1033 | 92.70% | 94.50% |
| 3 | normal | 90 | 353,629,537 | 84519 | 2907 | 4184 | 1048 | 85.20% | 87.70% |
| 3 | adenoma （1st load) | 90 | 390,700,094 | 94440 | 2443 | 4137 | 918 | 91.90% | 83.70% |
| 3 | adenoma（2nd load) | 90 | 447,910,735 | 168577 | 2785 | 2657 | 1017 | 90.90% | 89.20% |
| 3 | carcinoma | 90 | 384,614,660 | 146352 | 2379 | 2628 | 978 | 89.30% | 89.00% |

| **Table S3. Expression of BMX, SH2D6, REG4, and SOX4 on normal, adenoma, and carcinoma tissues.** | | | | | | | | | | |
| --- | --- | --- | --- | --- | --- | --- | --- | --- | --- | --- |
| **Patient** | **Gender** | **Age** | **BMX** | | **SH2D6** | | **REG4** | | **SOX4** | |
|  |  |  | Normal | Adenoma | Normal | Adenoma | Adenoma | Carcinoma | Adenoma | Carcinoma |
| 1 | F | 24 | - | - | + | + | ++ | ++ | + | + |
| 2 | M | 67 | - | + | - | + | + | + | + | + |
| 3 | F | 66 | - | + | - | ++++ | ++++ | ++ | + | + |
| 4 | M | 61 | + | + | + | + | ++ | ++++ | ++ | - |
| 5 | F | 76 | - | - | + | ++ | +++ | NA | ++ | NA |
| 6 | M | 72 | - | + | + | +++ | ++ | ++++ | +++ | + |
| 7 | F | 52 | ++ | ++ | + | +++ | +++ | ++++ | + | ++ |
| 8 | M | 62 | + | ++ | + | ++ | +++ | ++++ | ++ | +++ |
| 9 | M | 76 | + | + | - | + | - | ++ | + | + |
| 10 | F | 54 | - | - | ++ | - | ++ | + | ++ | ++ |
| 11 | F | 68 | + | + | + | ++ | +++ | ++ | ++ | ++ |
| 12 | F | 73 | - | + | + | +++ | ++ | +++ | +++ | - |
| 13 | M | 47 | + | ++ | + | ++ | + | ++ | ++ | ++ |
| 14 | F | 57 | + | - | - | ++ | ++++ | - | - | + |
| 15 | M | 69 | + | ++ | + | + | +++ | ++ | ++ | +++ |
| 16 | F | 68 | - | + | ++ | + | ++ | ++ | + | ++ |
| 17 | F | 71 | + | - | - | - | + | +++ | ++ | + |
| 18 | M | 42 | + | + | +++ | ++ | ++ | + | ++ | + |
| 19 | M | 69 | + | + | + | + | +++ | +++ | + | + |
| 20 | F | 57 | - | + | + | +++ | ++ | ++ | +++ | ++ |
| * NA, not available | | |  |  |  |  |  |  |  |  |

| **Table S4. Staining intensity for BMX, HCK, and MATK on epithelial cells in normal and adenoma tissues.** | | | | | | | | |
| --- | --- | --- | --- | --- | --- | --- | --- | --- |
| **Patient** | **Gender** | **Age** | **BMX** | | **HCK** | | **MATK** | |
|  |  |  | Normal | Adenoma | Normal | Adenoma | Normal | Adenoma |
| 1 | F | 24 | - | - | - | + | - | - |
| 2 | M | 67 | - | - | - | + | - | - |
| 3 | F | 66 | - | + | + | ++ | - | + |
| 4 | M | 61 | + | + | + | + | + | + |
| 5 | F | 76 | - | - | - | ++ | - | + |
| 6 | M | 72 | - | + | + | + | + | - |
| 7 | F | 52 | + | + | + | - | + | - |
| 8 | M | 62 | + | ++ | + | ++ | + | + |
| 9 | M | 76 | + | + | + | ++ | + | + |
| 10 | F | 54 | - | + | - | + | - | + |
| 11 | F | 68 | + | + | + | ++ | + | + |
| 12 | F | 73 | - | + | + | + | - | + |
| 13 | M | 47 | + | + | + | + | + | + |
| 14 | F | 57 | + | - | - | - | - | - |
| 15 | M | 69 | + | + | + | + | + | + |
| 16 | F | 68 | - | - | - | + | - | - |
| 17 | F | 71 | + | - | - | - | - | - |
| 18 | M | 42 | - | + | + | ++ | - | + |
| 19 | M | 69 | + | + | + | + | + | + |
| 20 | F | 57 | - | - | - | + | - | - |
|  | | |  |  |  |  |  |  |

**Data S1.**

DEGs of epithelial subtypes in P1 and P2.
